# Supplementary material for: Cost-effectiveness of rapid laboratory-based mass-spectrometry diagnosis of bloodstream infection: evidence from the RAPIDO randomised controlled trial
Source: BMJ Open. 2021 Oct 18;11(10):e044623. doi: 10.1136/bmjopen-2020-044623 (PMC8524273; doi:10.1136/bmjopen-2020-044623)
Supplement: Supplementary data [file bmjopen-2020-044623supp001.pdf]

SUPPLEMENTARY MATERIAL - Cost-effectiveness of rapid laboratory-based mass-spectrometry diagnosis of bloodstream infection: Evidence from the RAPIDO randomized controlled trial

Costing of hospital stays

The cost of ward stays was calculated from the 2012/13 NHS Reference Costs (1) and then inflated using Curtis and Burns (2) as described in main text.

Costing by ward speciality

Hospital admission was an inclusion criterion for the RAPIDO trial, but a small number of "non-admitted" ward-days could occur in cases where the diagnostic blood sample was taken in an outpatient or day-case setting shortly before admission to a more appropriate speciality. Therefore, all ward stays were costed as inpatients unless the ward speciality recorded in the dataset explicitly specified otherwise e.g. day-case surgery.

The ward specialities described in the dataset were mapped to their nearest equivalent Service Descriptions and currency codes in NHS Reference Costs as shown in Table A1. Where more than one Service Description or currency code applied to a ward speciality, the ward cost was calculated as an average of the unit costs of each Service Description or currency code, weighted by the number of bed-days reported for them in NHS Reference Costs. An example calculation is shown in Table A2.

The RAPIDO dataset did not include the number of organs supported in critical care so the cost of stays in high-dependency units (HDUs) and intensive therapy units (ITUs) was also calculated as a weighted average. The costs of a bed-day on an adult critical care unit for the different levels of organ support are listed in NHS Reference Costs; these costs were weighted by the number of bed-days reported for patients at each level of support to give an average cost for use in analysis.

Table A1 Matching of RAPIDO ward specialities to NHS Reference Cost categories

**RAPIDO ward speciality description**	**NHS Reference Cost 'Service Description'**	**NHS Reference Cost 'service code' or 'currency description'[a,b]**
Medical: Acute medical admissions and pre-admissions	Accident and Emergency	Admitted codes only[c]
Medical: Cardiology / cardiovascular / coronary	Cardiology	320
Medical: Care of the Elderly	Geriatric Medicine	430
Medical: Dermatology / rheumatology	Dermatology AND Rheumatology	410[R] AND 330[D]
Medical: Diabetes / endocrinology	Diabetic Medicine AND Endocrinology	307[D] AND 302[E]
Medical: Gastroenterology / gastrology / liver	Gastroenterology AND Hepatology	301 [G] and 306[H]
Medical: General medical (no declared speciality)	General Medicine	300
Medical: Haematology / oncology	Clinical haematology AND Clinical oncology	303[H] and 800[O]
Medical: Infectious disease / travel medicine	Infectious Diseases	350
Medical: Nephrology / renal / dialysis	Nephrology	361
Medical: Neurology / neurosciences / neuromedical	Neurology	400
Medical: Palliative	Palliative Medicine	315
Medical: Respiratory	Respiratory Medicine	328
Medical: Stroke	Stroke Medicine	340
ITU/HDU[d]: General (not specified as surgical, medical or specialist)	Adult Critical Care Unit	Critical care currencies
ITU/HDU: General medical	N/A[e]	N/A
ITU/HDU: General surgical	Adult Critical Care Unit	Critical care currencies
ITU/HDU: Cardiac	Adult Critical Care Unit	Critical care currencies
ITU/HDU: Neurology/neurosurgery	Adult Critical Care Unit	Critical care currencies
ITU/HDU: Renal	N/A	N/A
ITU/HDU: Theatre recovery areas	N/A	N/A
Surgery: Admissions / pre-admissions units	General surgery	100
Surgery: Cardiothoracic / thoracic	Cardiothoracic surgery AND Thoracic surgery	170[C] and 173[T]
Surgery: Ear, nose, throat, oral & maxillo-facial, and opthalmic units	All surgical codes[f]	Inpatient codes only[c]
Surgery: General including GI, breast, vascular	General surgery	100

Surgery: Neurosurgery	Neurosurgery	150
Surgery: Orthopaedic / trauma	Trauma & Orthopaedics	110
Surgery: Plastics / burns	Plastic surgery	160
Surgery: Short stay and daycase units	General surgery	100, day cases only
Surgery: Urology / renal	All surgical codes[f]	Inpatient codes only[c]
Other: A&E, emergency assessment, fracture clinics and related units	Accident and Emergency	Admitted codes only[c]
Other: Imaging, diagnostics and telemetry	N/A	N/A
Other: Obstetrics & gynaecology	Obstetrics	501
Other: Psychology / psychiatry / mental health	N/A	N/A
Other: Services - not medical, surgical or HDU/ITU, and not listed elsewhere	"Other Procedures or Health Care Problems"	Inpatient codes only[c]

Notes to Table A1:

[a] If there were separate codes for adults and children, only adult codes were used.

[b]If multiple NHS Service Descriptions or Currency Codes applied, costs were weighted by the number of bed-days occupancy for each Description or Code (or, in critical care, number of organs supported) reported in NHS Reference Costs.

[c] Only 'Inpatient' or 'Admitted' codes were used in these cases as all RAPIDO patients were admitted before or soon after taking of the diagnostic blood sample.

[d]HDU/ITU = critical care specialities (high dependency and intensive therapy units), costs weighted by number of bed-days occupancy for each number of organs supported in critical care.

[e] N/A = Not applicable – not costed as no observations in the trial.

[f]A weighted average across all admitted surgical codes was used for surgical specialities that were not listed specifically in NHS Reference Costs. Costing by length of stay

Hospitals in the NHS are paid according to patients' length of stay, with different levels of payment being made according to whether each bed-day of the stay is an 'inlier' bed-day or an excess (or 'outlier') bed-day. These payments constitute 'costs' from an NHS system perspective and we used them to cost hospital stays.

The total costs of the patients' stays in each arm of the trial depend on their whole ward history from the date of admission, not from the date of randomisation - although, clearly, differences due to the RAPIDO intervention could only accrue after randomisation. The total hospital cost could not be calculated because, although the date of admission was known, it was not known for how long patients had been on particular wards before the diagnostic blood samples were taken.

The distinction between an inlier bed-day and an excess bed-day is defined by reference to a trim point, a figure which is intended to capture the upper end of the range of lengths of stay expected for a given currency code. It is calculated as the (upper quartile of length of stay) + 1.5 × (interquartile range of length of stay). In a long stay, days after the trim point are counted as excess bed-days, while days up to the trim point are counted as inliers.

For inlier costs, national average unit costs and average length of stay (number of days) per Finished Consultant Episode are reported. A Finished Consultant Episode is a completed episode of treatment

received by a patient under the care of one consultant. Dividing the mean nationwide unit cost for a given currency code by the mean nationwide number of inpatient days for a patient admitted under that currency code gives its estimated per-day cost. To obtain the weighted cost for each currency code, we multiply the per-day cost by the share of that currency code in the total bed-days of all currency codes in each Service Description. The sum of these figures is the weighted average per-day cost for either elective inpatient or non-elective inpatients receiving treatment under that Service Description. Unit costs per excess bed day are calculated in a similar manner.

Elective and non-elective stays

The RAPIDO dataset did not include information to classify each day of each patient's stay in a ward as elective or non-elective. It is likely that a high proportion of ward admittances after day 0 of the trial were non-elective since they followed a presumed diagnosis (or, at least, clear suspicion) of bloodstream infection, as evidenced by the taking of a blood sample for culture. However, it is possible that the infection developed during an elective admission, so ward costs – particularly on day 0 – might in fact be charged as elective. We therefore included both elective and non-elective costs in our calculations, by weighting the average cost of all currency codes in each category (elective and non-elective) by their respective shares in bed-days.

A post hoc analysis examined the sensitivity of unit costs to the exclusion of elective care. The effect on unit costs of this exclusion was modest. In some cases, unit costs do not distinguish between elective and non-elective (e.g. for critical care) and the exclusion had no effect, and in other cases the effect of excluding elective admissions was to reduce unit costs by approximately 0.1% to 1%. We consider that including elective care in the calculation of unit costs in general is a conservative approach, and one appropriate to our base-case analysis. We therefore did not re-run this analysis using the very slightly lower unit costs that would have arisen under the exclusion of elective care.

Example: calculation of per-day cost for a 'General Medical' ward stay

The steps involved in the calculation of unit costs for the 'General Medicine' Service Description are set out in Table A2.

Table A2 Example calculation of weighted per-day cost associated with the 'General Medicine' Service Description in 2012/13

Item	Quantity or share of total	Calculation
Number of bed-days[a]		
National total bed-days per year: Elective Inpatient excluding short-stay (EI)	96,339	A
National total bed-days per year: Non-Elective Inpatient (NEI)	6,398,877	B
National total bed-days per year: Non-Elective Inpatient Short-stay (NEI-S)	1,545,596	C
National total bed-days per year: Inpatients (EI + NEI + NEI-S)	8,040,812	D=(A+B+C)
Share of bed-days		

% of days that relate to EI	1.20%	E=A/D
% of days that relate to NEI	79.58%	F=B/D
% of days that relate to NEI-S	19.22%	G=C/D
Averaging over inlier/outlier bed-days and currency codes		
Weighted[b] average per-day cost: EI	£456.18	H[b]
Weighted[b] average per-day cost: NEI	£327.48	I[b]
Weighted[b] average per-day cost: NEI-S	£434.19	J[b]
Contributions of elective, non-elective and short stays		
Weighted[c] EI cost per day	£5.47	K=H × E
Weighted[c] NEI cost per day	£260.61	L=F × I
Weighted[c] NEI-S cost per	£83.46	M=G × J
Estimated mean cost per day for RAPIDO analysis		
Cost of a General Medical ward-day for RAPIDO analysis i.e. weighted average of EI, NEI and NEI-S per day costs	£349.53	K+L+M

[a]'Bed-days' here captures both bed days and excess bed days. [b]These figures are calculated as the average of bed-day-weighted sum of costs for inlier bed-days and excess bed-days across all currency codes within the 'General Medicine' service description, weighted by proportion of bed-days from each currency code, as described in the text. [c]These figures are weighted by the proportion of bed-days from each type of inpatient stay (elective, non-elective, and non-elective short-stay).

Costing of MALDI-TOF spectrometry

Data from published literature and confidential information provided by one study centre was used to estimate a mean per blood-sample cost of diagnosis using MALDI-TOF technology at 2012/13 prices and then subsequently inflated.

At this centre, 4,303 machine-positive adult blood samples were recorded during the whole study period, equating to 2,061 per year. In routine use of the technology, all of these would have been tested by MALDI-TOF but fewer were actually tested during the RAPIDO trial. Only 3,153 flagged positive during study hours (=1510/year) and, with 1:1 randomisation, only approximately half of those (755) would have been allocated to MALDI-TOF diagnosis.

We estimated the proportion of total MALDI-TOF cost attributable to use for positive blood cultures as 12% based on two considerations. First, approximately 10-15% of all microbiology laboratory requests for 'culture and sensitivity' related to blood samples. Second, at this centre, organisms from blood accounted for approximately 12% of all organisms identified.

We used these figures to calculate the capital, operating, labour and consumable costs used per positive sample as set out in Table A3. Salary costs and information concerning on costs and overheads were taken from Curtis (2013).(3)

Table A3 Calculation of unit cost of MALDI-TOF identification in 2012/13 prices

Item	Mean value	Comment / data source
Capital-related costs		
Capital cost for new MALDI-TOF machine	£130,000	Published literature, catalogue prices, confidential information from one study centre
Economic life used for depreciation calculations	10 years	Published literature, catalogue prices, confidential information from one study centre
Annual capital charge per blood sample	£0.76	
Maintenance costs		
Annual non-reagent maintenance contract (13% of capital cost)	£16,900	Published literature, confidential information from one study centre
Maintenance cost per blood sample	£0.98	
Consumable costs		
Consumables cost per blood sample, as used in RAPIDO protocol	£0.17	Trial protocol, catalogue data, information from one study centre
Labour costs, including on-costs and overheads		
Band 5 salary	£62,927	Staff grades at one study centre, published NHS payscales, Curtis (2013) for information on oncosts and overhead
Band 6 salary	£76,569	Staff grades at one study centre; published NHS payscales; information on on-costs and overheads from Curtis (2013).(3)
Labour cost per sample	£4.17	...assuming that each grade contributes equally to processing of all blood samples
Total cost		
Total cost per machine-positive blood sample analysed with MALDI-TOF	£6.08	

CONSORT diagram of patient numbers

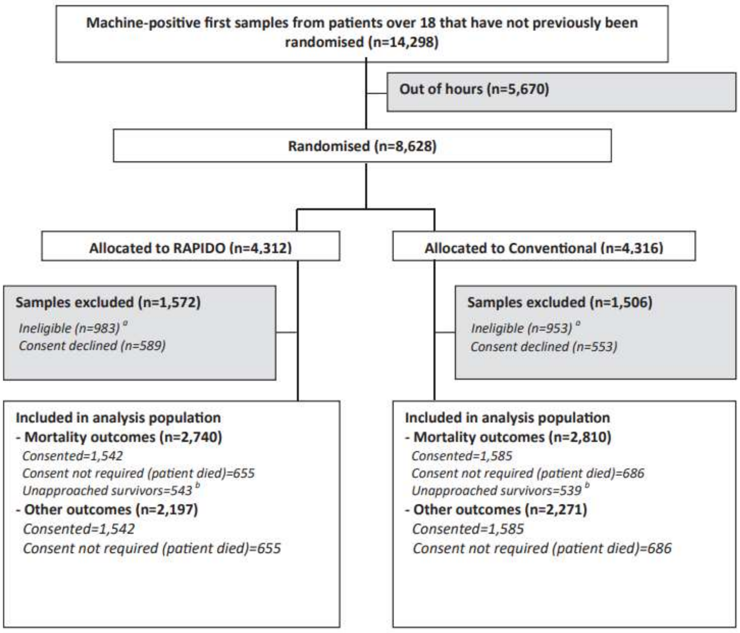

Notes to figure: Flow of patients. a Ineligible samples include 111 rapid diagnosis and 125 conventional samples that were randomized in error, and 872 rapid diagnosis and 828 conventional that were randomized correctly but met postrandomization exclusion criteria. b Unapproached survivors are eligible patients who could not be approached for consent, usually because of lack of capacity and inability to identify a consultee. They are included in mortality analysis (only) as 28-day survivors.

Costs and results in 2012/13 price levels

Table A4 Costs in available cases

Mean cost	Control (N=2,271)	RAPIDO (N=2,197)	Difference (95% CI) [a]
Intervention cost	-	£6	+£6
Antimicrobial cost	£247	£265	+£18 (-£6 to £41)
7-day ward costs	£3,448	£3,404	-£44 (-£165 to £77)
Total 7-day costs	£3,695	£3,675	-£20 (-£148 to £108)
28-day ward costs	£8,451	£8,412	-£39 (-£505 to £427)
Total 28-days costs	£8,698	£8,682	-£15 (-£487 to £456)

***Note**: [a] Confidence intervals around mean differences calculated from unadjusted linear regression*

Cost-effectiveness and sensitivity analysis

In the base-case imputed analysis, estimated mean costs per patient were lower in the RAPIDO arm (mean difference -£114; 95% CI:-£710 to £482), and the proportion of patients alive at day 28 was also lower (81.4% vs 82.3%, see Table A5). Ward costs, including the costs of conventional microbiological testing, constituted 97% of total costs in each arm. Most of the remaining 3% of total cost was attributable to antimicrobial costs. The estimated per-patient cost of diagnosis using MALDI-TOF (£6.08) constituted a negligible proportion of overall per-patient mean costs in the intervention arm.

Table A5 Costs and outcome: base-case analysis with imputation (N=5,550)

	Control	RAPIDO	Difference (95% CI)
Mean 28-day NHS costs	£ 7,485	£7,371	-£114 (-£710 to £482)
28-day survival[a]	0.823	0.814	-0.009 (-0.029 to 0.011)

***Note:** [a]Survival measured as the proportion of patients alive at day 28.*

Table A6 Cost-effectiveness: base-case analysis with imputation (N=5,550)

Threshold[a]	Net monetary benefit (95% CI)	Probability of cost-effectiveness
£5,000	£71 (-£519 to £661)	0.59
£10,000	£28 (-£579 to £636)	0.54
£20,000	-£57 (-£745 to £630)	0.44
£30,000	-£143 (-£954 to £668)	0.36
£50,000	-£315 (-£1,443 to £814)	0.29

***Note:** [a]Threshold value = 28-day cost per death avoided at 28 days.*

The probability of the RAPIDO intervention being cost-effective declines with increasing threshold values of cost per death avoided at 28 days, as shown in Figure A1.

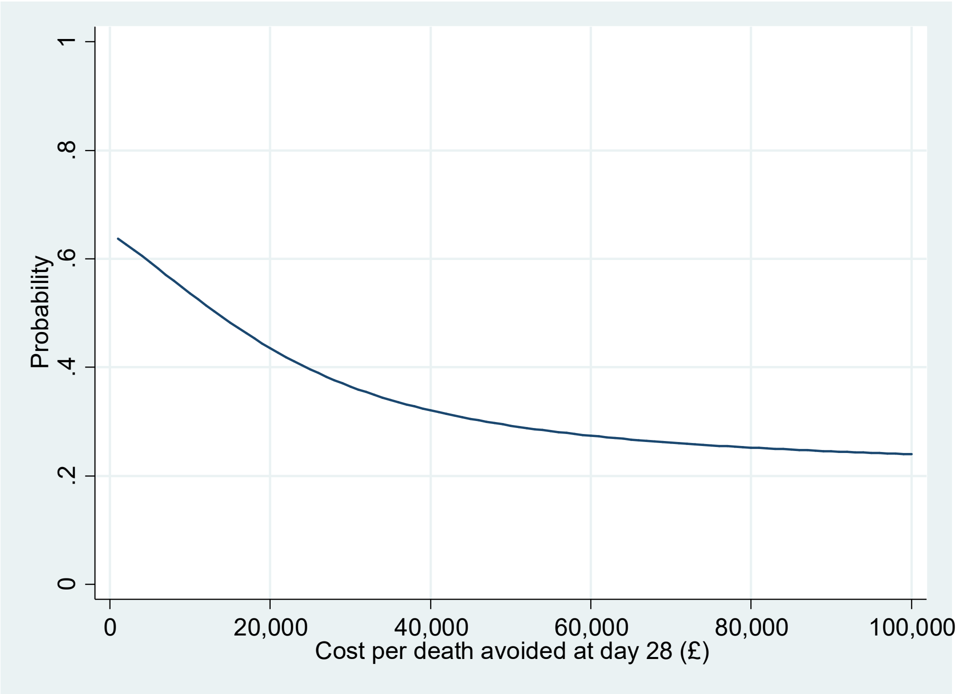

Figure A1 Cost-effectiveness acceptability curve for base case

Table A7 and A8 report the results of the various sensitivity analyses. These analyses, expressed as net monetary benefit (with associated 95% confidence intervals), do not differ substantially from the base-case results. Estimating costs at seven rather than 28 days did not alter the overall cost-effectiveness conclusions.

Subgroup analysis

Table A9 and Table A10 present the results of the subgroup analysis comparing clinically significant and clinically non-significant episodes of bloodstream infection. Statistical tests for interaction showed no evidence of a subgroup effect (p-value for interaction in the cost seemingly unrelated regression equation=0.32, p-value in the outcome seemingly unrelated regression equation =0.66), and estimates of difference between Conventional and RAPIDO diagnosis in both outcome and costs were broadly similar for the clinically significant and clinically non-significant subgroups.

Table A7 Sensitivity analysis: costs and outcome

		Excluding unapproached survivors, N=4,468, cost at 28 days		Excluding unapproached survivors, N=4,468, cost at 7 days		Including unapproached survivors, N=5,550, cost at 7 days	
NHS costs mean (95% CI)	Control	£8,705	(£8,376 to £9,033)	£3,697	(£3,608 to £3,786)	£3,324	(£3,098 to £3,598)
	RAPIDO	£8,675	(£8,341 to £9,009)	£3,673	(£3,583 to £3,763)	£3,238	(£3,094 to £3,619)
	Difference	-£30	(-£498 to £439)	-£24	(-£151 to £103)	-£86	(-£324 to £153)
28-day survival mean (95% CI)	Control	0.78	(0.76 to 0.80)	0.78	(0.76 to 0.80)	0.82	(0.81 to 0.84)
	RAPIDO	0.77	(0.75 to 0.79)	0.77	(0.75 to 0.79)	0.81	(.80 to 0.83)
	Difference	-0.01	(-0.04 to 0.01)	-0.01	(-0.04 to -0.01)	-0.01	(-0.03 to 0.01)

Table A8 Sensitivity analysis: cost effectiveness

	Excluding unapproached survivors, N=4,468, cost at 28 days			Excluding unapproached survivors, N=4,468, cost at 7 days			Including unapproached survivors, N=5,550, cost at 7 days		
Threshold[a]	NMB	(95% CI)	PCE[b]	NMB	(95% CI)	PCE	NMB	(95% CI)	PCE
£50,000	-£32	(-£489 to £425)	0.45	-£38	(-£199 to £124)	0.32	£43	(-£209 to £295)	0.63
£10,000	-£94	(-£572 to £385)	0.35	-£99	(-£356 to £158)	0.23	£0	(-£305 to -£305)	0.50
£20,000	-£217	(-£814 to £381)	0.24	-£222	(-£707 to £263)	0.18	-£86	(-£548 to £377)	0.36
£30,000	-£340	(-£1,117 to £438)	0.20	-£345	(-£1,069 to £379)	0.17	-£171	(-£817 to £474)	0.30
£50,000	-£586	(-£1,793 to £622)	0.17	-£591	(-£1,799 to £616)	0.17	-£343	(-£1,376 to £691)	0.26

Notes: *[a]Threshold value = cost per death avoided at 28 days; [b]NMB = net monetary benefit [mean (95% confidence interval)]; [c]PCE = probability of cost-effectiveness at given threshold.*

Table A9 Subgroup analysis: costs and outcome

		All (unapproached survivors excluded) N=4,468		Clinically significant episodes only N=3,010 (67%)		Clinically non-significant episodes only N=1,458 (33%)	
28-day NHS costs mean (95% CI)	Control	£8,705	(£8,376 to £9,033)	£8,570	(£8,148 to £8,992)	£8,565	(£7,962 to £9,168)
	RAPIDO	£8,675	(£8,341 to £9,009)	£8,716	(£8,278 to £9,155)	£8,199	(£7,611 to £8,787)
	Difference	-£30	(-£498 to £439)	£146	(-£425 to £717)	-£365	(-£1,186 to £454)
28-day survival mean (95% CI)	Control	0.78	(0.76 to 0.80)	0.78	(0.76 to 0.79)	0.80	(0.77 to 0.83)
	RAPIDO	0.77	(0.75 to 0.79)	0.77	(0.75 to 0.79)	0.78	(0.75 to 0.81)
	Difference	-0.01	(-0.04 to 0.01)	-0.01	(-0.04 to 0.02)	-0.02	(-0.06 to 0.02)

Table A10 Subgroup analysis: cost effectiveness at 28 days

Threshold value[a]	All (unapproached survivors excluded) N=4,468			Clinically significant episodes only N=3,010 (67%)			Clinically non-significant episodes only N=1,458 (33%)		
	NMB[b]	(95% CI)	PCE[c]	NMB	(95% CI)	PCE	NMB	(95% CI)	PCE
£50,000	-£32	(-£489 to £425)	0.45	-£191	(-£780 to £390)	0.26	£263	(-£585 to £1,110)	0.73
£10,000	-£94	(-£572 to £385)	0.35	-£235	(-£879 to £409)	0.24	£160	(£765 to £1,085)	0.63
£20,000	-£217	(-£814 to £381)	0.24	-£324	(-£1,149 to £501)	0.22	-£47	(-£1,231 to £1,138)	0.47
£30,000	-£340	(-£1,117 to £438)	0.20	-£413	(-£1,472 to £647)	0.22	-£253	(-£276 to -£230)	0.37
£50,000	-£586	(-£1,793 to £622)	0.17	-£591	(-£2,814 to £1,003)	0.23	-£665	(-£700 to -£631)	0.28

[a]Threshold value = cost per death avoided at 28 days; [b]NMB = net monetary benefit [mean (95% confidence interval)]; [c]PCE = probability of cost-effectiveness at given threshold.

References

1. **Department of Health** (2013) Reference Costs 2012/13. London.

2. **Curtis L & Burns A** (2018) Unit Costs of Health and Social Care 2019. Personal Social Services Research Unit. University of Kent, Canterbury.

3. **Curtis L** (2013) *Unit Costs of Health and Social Care 2013*. Canterbury: Personal Social Services Research Unit.
