# Supplementary material for: Cost-effectiveness of rapid laboratory-based mass-spectrometry diagnosis of bloodstream infection: evidence from the RAPIDO randomised controlled trial
Source: BMJ Open. 2021 Oct 18;11(10):e044623. doi: 10.1136/bmjopen-2020-044623 (PMC8524273; doi:10.1136/bmjopen-2020-044623)
Supplement: Author's manuscript [file bmjopen-2020-044623.draft_revisions.pdf]

BMJ Open

BMJ Open is committed to open peer review. As part of this commitment we make the peer review history of every article we publish publicly available.

When an article is published we post the peer reviewers' comments and the authors' responses online. We also post the versions of the paper that were used during peer review. These are the versions that the peer review comments apply to.

The versions of the paper that follow are the versions that were submitted during the peer review process. They are not the versions of record or the final published versions. They should not be cited or distributed as the published version of this manuscript.

BMJ Open is an open access journal and the full, final, typeset and author-corrected version of record of the manuscript is available on our site with no access controls, subscription charges or pay-per-view fees (http://bmjopen.bmj.com).

If you have any questions on BMJ Open's open peer review process please email info.bmjopen@bmj.com

BMJ Open

Cost-effectiveness of rapid laboratory-based mass-spectrometry diagnosis of bloodstream infection: Evidence from the RAPIDO randomized controlled trial

Journal:	*BMJ Open*
Manuscript ID	bmjopen-2020-044623
Article Type:	Original research
Date Submitted by the Author:	10-Sep-2020
Complete List of Authors:	Dixon, Padraig; Oxford University,<br>Hollingworth, William; University of Bristol, School of Social and Community Medicine<br>Pike, Katie; Bristol University<br>Reynolds, Rosy; Bristol Medical School<br>Stoddart, Margaret; Southmead Hospital<br>MacGowan, Alasdair; Southmead Hospital
Keywords:	HEALTH ECONOMICS, Diagnostic microbiology < INFECTIOUS DISEASES, Infection control < INFECTIOUS DISEASES

SCHOLARONE™
Manuscripts

I, the Submitting Author has the right to grant and does grant on behalf of all authors of the Work (as defined in the below author licence), an exclusive licence and/or a non-exclusive licence for contributions from authors who are: i) UK Crown employees; ii) where BMJ has agreed a CC-BY licence shall apply, and/or iii) in accordance with the terms applicable for US Federal Government officers or employees acting as part of their official duties; on a worldwide, perpetual, irrevocable, royalty-free basis to BMJ Publishing Group Ltd ("BMJ") its licensees and where the relevant Journal is co-owned by BMJ to the co-owners of the Journal, to publish the Work in this journal and any other BMJ products and to exploit all rights, as set out in our licence.

The Submitting Author accepts and understands that any supply made under these terms is made by BMJ to the Submitting Author unless you are acting as an employee on behalf of your employer or a postgraduate student of an affiliated institution which is paying any applicable article publishing charge ("APC") for Open Access articles. Where the Submitting Author wishes to make the Work available on an Open Access basis (and intends to pay the relevant APC), the terms of reuse of such Open Access shall be governed by a Creative Commons licence – details of these licences and which Creative Commons licence will apply to this Work are set out in our licence referred to above.

Other than as permitted in any relevant BMJ Author's Self Archiving Policies, I confirm this Work has not been accepted for publication elsewhere, is not being considered for publication elsewhere and does not duplicate material already published. I confirm all authors consent to publication of this Work and authorise the granting of this licence.

For peer review only

Cost-effectiveness of rapid laboratory-based mass-spectrometry diagnosis of bloodstream infection: Evidence from the RAPIDO randomized controlled trial

Padraig Dixon[1]*, William Hollingworth[2], Katie Pike[3], Rosy Reynolds[2], Margaret Stoddart[4], Alasdair MacGowan[4,5]

1 Nuffield Department of Primary Care Health Sciences, University of Oxford

2 Population Health Sciences, University of Bristol

3 Bristol Trials Centre (Clinical Trials and Evaluation Unit), University of Bristol

4 Infection Sciences, North Bristol NHS Trust, Bristol

5 School of Cellular and Molecular Medicine, University of Bristol

*Corresponding author

Email: Padraig.Dixon@phc.ox.ac.uk

Short title: Cost-effectiveness of rapid bacteraemia identification

Funding statement: This report summarises independent research funded by the National Institute for Health Research (NIHR) under its Programme Grant for Applied Research (PGfAR RP-PG-0707-10043). The views and opinions expressed in this report are those of the authors and do not necessarily reflect those of the National Institute for Health Research (NIHR), the NHS or the Department of Health. The funder had no role in the conduct of the study, the writing of the manuscript or the decision to submit it for publication.

Acknowledgements: We are very grateful to all patients, health care professionals, and NHS staff who contributed time and effort to make the RAPIDO trial possible. We are grateful to administrative staff at trial sites for support with participant recruitment, data entry and trial administration. The RAPIDO trial was designed and delivered in collaboration with Clinical Trials

For peer review only

and Evaluation Unit Bristol (CTEU), a UKCRC Registered Clinical Trials Unit in receipt of National Institute for Health Research CTU support funding. RR acknowledges support from the NIHR Health Protection Research Unit in Behavioural Science and Evaluation at University of Bristol.

Conflict of interest statement: The authors declare no conflicts of interest.

Ethics: The RAPIDO trial was approved by the NRES Committee South West - Frenchay on 20 March 2012, reference 12/SW/003.

Informed consent: In order to initiate rapid diagnosis quickly, the study design required prompt randomisation when blood cultures flagged positive, so it was necessary to seek consent retrospectively. Further details are provided in the main text and in references to the main trial publication.

Key words: Bloodstream infection, randomised controlled trial, MALDI-TOF, mass spectrometry, cost-effectiveness.

Word count: 3,953

The differences between arms in cost and effect were modest, associated with uncertainty, and may not accurately reflect “real-world” routine use of MALDI-TOF technology in this patient group.

For peer review only

Abstract

Objectives and intervention

Bloodstream infection, the presence of viable microorganisms in the blood, is a prevalent clinical event associated with substantial mortality. Patient outcomes may be improved when the causative microorganism is identified quickly. We assessed the cost-effectiveness of rapid microbial identification by matrix-assisted laser desorption/ionisation time-of-flight (MALDI-TOF) mass spectrometry.

Design

Economic evaluation alongside a randomised multicentre trial (RAPIDO: RAPId Diagnosis on Outcome) assessing the impact of rapid identification by MALDI-TOF spectrometry.

Setting

Adult in-patients with bloodstream infections at seven NHS hospital trusts in England and Wales.

Primary outcome

Net monetary benefit, estimated as incremental costs compared with incremental 28-day survival, of rapid identification by MALDI-TOF spectrometry compared to conventional identification.

Methods

Patients were randomised (1:1) to receive diagnosis by conventional methods of microbial identification (conventional arm) only or by MALDI-TOF spectrometry in addition to conventional identification (RAPIDO arm).

Results

Data from 5,550 patients were included in primary analysis. Mean imputed costs in 2018/19 prices per patient were lower by £126 in the RAPIDO arm (95% CI: -£784 to £532) but the proportion of patients alive at day 28 was lower (81.5% versus 82.3%). The probability of cost-

For peer review only - http://bmjopen.bmj.com/site/about/guidelines.xhtml

effectiveness of MALDI-TOF was <0.5 at cost-effectiveness thresholds between £20,000 and £50,000.

Conclusions

Adjunctive MALDI-TOF diagnosis was unlikely to be cost-effective when measured as cost per death avoided at 28 days. However, the differences between arms in cost and effect were modest, associated with uncertainty, and may not accurately reflect "real-world" routine use of MALDI-TOF technology in this patient group.

Trial registration/reference: ISRCTN 97107018 / UKCRN 11978

Strengths and limitations of this study

We report an economic evaluation of the first randomized controlled trial of adjunctive matrix-assisted laser desorption/ionisation time of flight (MALDI-TOF) mass spectrometry identification of the causative microorganism in bloodstream infection.

Data on 5,550 patients from the RAPIDO (RAPId Diagnosis on Outcome) trial were used to estimate the cost-effectiveness of MALDI-TOF in comparison to conventional microbiological methods.

The intervention was unlikely to be cost effective, but data on quality-adjusted life years and long-term data beyond 28 days were not available.

For peer review only

Background

Bloodstream infection refers to the presence, confirmed by laboratory testing, of viable microorganisms in the bloodstream. These infections are both prevalent and clinically significant. There are estimated to be approximately 1.2 million annual episodes of bloodstream infection in Europe, 500,000 in North America [1], and100,000 in England and Wales [2]. Estimates of overall mortality range from 15-25% at 30 days post-infection to almost 50% at 12-36 months after infection [3-6].

Rapid identification of the causative microbial pathogen may be associated with improved patient outcomes [7 8]. The RAPIDO trial assessed the impact of laboratory-based RAPId Diagnosis on Outcome of bloodstream infections in hospitalised adult patients at seven NHS Hospital Trusts in England and Wales [2]. Rapid diagnosis was by matrix-assisted laser desorption/ionisation time-of-flight (MALDI-TOF) mass spectrometry applied to machine-positive blood cultures. Here we report the results of an economic evaluation of this trial, from an NHS perspective. Its purpose was to estimate the cost-effectiveness of using MALDI-TOF technology in addition to conventional microbiological techniques compared to conventional approaches alone.

Methods

Trial methods were described in MacGowan et al [2] and are summarised here.

Design

RAPIDO was a multicentre prospective randomised (1:1) non-blinded parallel-group trial comparing two approaches to identification of the causative microorganism(s) of bloodstream infection in hospitalised adult patients at seven centres in England and Wales. The primary outcome was 28-day survival, and the two approaches were MALDI-TOF spectrometry in addition to conventional microbiological culture (‘RAPIDO’ arm) or conventional culture only (‘Conventional’ arm).

Setting and participants

Adult patients aged ≥18 years, admitted to hospital for NHS care, and with a blood sample culture-positive for bacteria or fungi were potentially eligible for inclusion, whether or not the

For peer review only - http://bmjopen.bmj.com/site/about/guidelines.xhtml

organisms were considered clinically significant. Patients were not eligible if they were on an end-of-life pathway, had been previously randomised in the study, were prisoners or young offenders in the custody of the prison service, if the attending physician deemed them unsuitable, or if the positive blood culture entered the diagnostic pathway ‘out of hours’ when both MALDI-TOF and conventional identification methods were not equally available for use. Patients were randomised on a 1:1 basis to either the Conventional or RAPIDO arm.

In order to initiate rapid diagnosis quickly, the study design required prompt randomisation when blood cultures flagged positive, so it was necessary to seek consent retrospectively. Research nurses approached patients for consent when they were sufficiently recovered and had capacity, in the opinion of both the clinical team and the research nurse. If a patient did not have capacity, but was thought likely to regain it, then nurses waited for capacity to return. Otherwise, a relative or close friend of the patient was approached as consultee, if available. If patients with capacity were discharged to independent living before consent could be obtained, consent was sought by post.

Patient and public involvement

The public and patient panel involvement group for microbiology at North Bristol NHS Trust was consulted on study design and the material given to patients.

Intervention

Samples in both arms of the trial were tested by the conventional methods in routine use at the microbiology laboratory of the centre concerned, starting with incubation in a blood culture machine. Each laboratory’s standard operating procedures defined the choice of appropriate biochemical tests and antimicrobial panels, depending on all the information about the organism available at the time.

In the RAPIDO arm, samples were first also tested by MALDI-TOF mass spectrometry, which is a relatively new technology for the identification of microbial organisms.[9] Identification may be achieved within minutes, a much shorter time than for conventional identification.[9]

For peer review only

Data collection and outcomes

Research nurses and laboratory staff collected data on paper data capture forms for later entry to a web-based database. Detailed data collection lasted from day 0 to day 7 after blood sampling, or discharge or death if earlier, and continued until day 28 for the key outcomes of death, discharge and *C. difficile* infection, and for laboratory data if necessary.

Key clinical data relevant to the economic evaluation included dates of admission, blood sampling (date 0), discharge and death, allowing calculation of duration of stay both before and up to 28 days after the onset of bloodstream infection. All relevant antimicrobial prescriptions were recorded from day 0 to day 7 including drug names, doses, routes and frequencies of administration, and the number of doses actually taken on each day. Ward speciality was recorded for the ward where the patient spent most of each day up to day 7.

For the economic analysis, the trial's survival outcome was expressed as the proportion of patients alive at 28 days, so that the cost-effectiveness results could be interpreted as the incremental cost per percent change in the proportion of patients alive at 28 days.

Measurement and valuation of resource use

The economic analysis took a health system (i.e. NHS) secondary-care perspective for costs. The time horizon for the economic analysis was up to 28 days. Costs and outcomes were therefore not discounted. Costs were first calculated in 2012/13 prices to reflect the structure of relative costs within the NHS during the first year of participant recruitment into the RAPIDO RCT. These costs were then inflated to 2018/19 levels. Costs from 2012/13 to 2015/2016 were inflated using the hospital and community health services (HCHS) index [10]. This index was replaced in 2016 by the NHS cost inflation index (NHSCII) which was used to inflate prices from 2015/16 to the 2018/19 prices. Supplementary material contains results for the uninflated 2012/13 price levels.

The measured components of NHS costs in each arm were: diagnostic testing (reflecting differences in the technology randomly allocated for sample diagnosis); length of stay for initial

For peer review only - http://bmjopen.bmj.com/site/about/guidelines.xhtml

admission and ward type (reflecting different levels of intensity of clinical support); and antimicrobials prescribed. Further details are available in the supplementary material.

Diagnostic testing

Patients in both arms of the study had conventional diagnostic blood testing. The costs of the conventional approach to diagnosis are 'bundled' within NHS reference cost and tariffs categories for hospital admissions. To avoid double counting of these costs, the costs of conventional diagnosis were not separately calculated. The unit cost of identifying an organism directly from a machine-positive blood sample using MALDI-TOF was calculated using a microcosting exercise, described further in the supplementary material.

Length of stay and ward type

Data on the speciality of the ward on which patients were located was recorded up to the seventh day after the positive blood sample was taken. Unit costs by ward type are not provided in national data sources such as NHS Reference Costs.[11] The closest analogue in NHS Reference Costs is that of 'Service Description', which groups together related procedures. The coded ward specialities were therefore matched to the closest category of 'Service Description' contained in NHS Reference Costs.

For each Service Description, we calculated a per-day cost as the average of the costs for relevant currency codes (which combine patients with similar cost implications) weighted by the frequency of codes as reported in NHS Reference Costs. We also accounted for the remuneration of hospitals according to the length of stay of patients, and for differences in elective and non-elective care. Further details are provided in the supplementary information section.

We estimated ward costs between day seven (the last day at which ward type was recorded) and day 28 (the point at which the primary outcome of the RCT was measured) by a simple extrapolation. This involved assuming that, for those patients known to survive to at least day 7, that the day 7 ward type was the ward type on which patients were located until the earliest of

For peer review only

discharge, death or day 28. We assess the sensitivity of the results to this assumption by comparing the primary outcome to costs at 7 days.

Antimicrobial use

Antimicrobial drugs administered were costed to 2012/13 prices using the British National Formulary [12] and then inflated as with other costs to 2018/19. A per-patient, per-day antimicrobial cost was calculated from the recorded prescriptions and number of doses taken each day.

Analytical methods

We adopted an intention-to-treat approach to analysis, in which all costs and outcomes were analysed according to the diagnostic pathway to which participants' samples were randomised, rather than the pathway actually followed. All analysis was conducted using Stata version 15.1 (StataCorp, College Station, Texas, USA).

Imputation of missing data

The amount of missing data was modest for patients who provided consent. Mortality data at 28 days was available for all but two patients. Information on allocation was complete. Data necessary to cost ward stays was incomplete in 4% of cases, and in 12% of cases for antimicrobials. Logistic regression analysis confirmed that for each cost category this missingness was unrelated to randomised allocation; odds ratios (95% confidence intervals) for missing ward and antimicrobial data were 1.09 (0.80 to 1.49) and 0.93 (0.79 to 1.09), respectively. We used mean imputation, stratified by allocation, in order to include these data points in the complete case analysis.

Our base-case analysis used multiple imputation. Of the 5,550 patients in the analysis population, 19.5% (n=1,082) were eligible but unapproached survivors. Only very limited baseline information was available for these patients, in accordance with the ethical approvals received, but their mortality outcome was known. Our base-case analysis used multiple imputation to estimate the 28-day and 7-day costs of the 1,082 unapproached survivors.

For peer review only

Multiple imputation by chained equations was implemented in Stata 15.1 using the –ice – command.[13 14] The imputation model was stratified by trial arm and included all variables for which there was complete information on the 5,550 analysis sample patients (centre, sex, age, and consent status) and total cost at 7 and 28 days for available cases. We also included the 28-day mortality outcome. We assumed that the two patients for whom the mortality outcome was censored before day 28 had died by day 28. Predictive mean matching[13] was used to allow for non-normal distributions of the cost variables. Costs were imputed at the level of 7- and 28-day costs, rather than for the underlying disaggregated components of these costs. The number of imputations (n=30) was selected to be larger than 100 times the proportion of missing data.[13] We reflected variation within and between the imputed datasets in the analysis using the methods described by Faria et al.[15]

Cost-effectiveness analysis

Cost-effectiveness parameters were calculated parametrically from the output of seemingly unrelated regressions in which NHS costs and then the proportion of patients alive at 28 days were regressed on a binary treatment indicator and indicators for trial centre.

In the absence of a survival or mortality-specific threshold, we estimated net monetary benefit (NMB) at a range of threshold values (£5,000, £10,000, £20,000, £30,000 and £50,000 per death avoided at 28 days). To quantify uncertainty, we calculated confidence intervals around point estimates of net benefit, and constructed cost-effectiveness acceptability curves.

We undertook a number of sensitivity analyses. We calculated net benefit excluding the group of eligible but unapproached survivor patients. We also calculated cost-effectiveness at seven days (rather than 28) with and without this group of patients in order to assess whether our findings in the base-case were substantially affected by our extrapolation of ward costs beyond day 7 for participants surviving to this point.

Subgroup analysis

We undertook one pre-specified subgroup analysis to examine the clinical significance of the infection episode. A positive blood culture may reflect the presence of pathogenic organisms

For peer review only

multiplying in the patient's bloodstream (clinically significant infection), or an incidental contamination of the blood sample during blood sampling or laboratory processing (not clinically significant).

We followed the regression-based methods for subgroup analysis set out by Willan et al.[16] by introducing a factorial subgroup/allocation interaction into the cost and effect equations. This analysis necessarily excluded the unapproached survivor group for whom information on clinical significance was unavailable.

Results

A total of 14,298 samples were presented for screening as first machine-positive samples from adult patients during the study period. From this total, 5,670 samples were excluded as occurring out-of-hours and the remaining 8,628 samples were randomised to either RAPIDO (n=4,312) or Conventional identification (n=4,316). Excluding those who were ineligible or declined consent resulted in an analysis population of 5,550 patients (2,740 RAPIDO, 2,810 Conventional). An unexpectedly large group of patients survived to at least day 28 but were not approached for consent because they lacked capacity and no suitable consultee could be found for them. The analysis population of 5,550 included 1,082 eligible but unapproached survivors (543 RAPIDO, 539 Conventional).

Outcomes

A slightly higher proportion of patients died by 28 days in the RAPIDO group (18.5% or 508/2,470) than in the Conventional group (17.7% or 497/2,810). The hazard ratio (calculated from Cox proportional hazards regression) was 1.05 (95% CI: 0.93 to 1.19, p=0.42). Median time to discharge (up to 28 days) was 15 days in both arms (hazard ratio=0.98; 95% CI: 0.90 to 1.06).

There was limited deviation from the protocol with respect to diagnostic pathway: the correct diagnostic pathway was not followed for 6.1% of patients in the intervention arm (133/2196) and 2.1% in the control arm (48/2271).

For peer review only

Costs

Costs from available cases, after mean imputation but before multiple imputation, are presented in *Table 1*. Costs are similar between arms, with the intervention having slightly lower total costs (the sum of intervention, antimicrobial and ward costs) at 7 and 28 days.

Table 1 Costs in available cases

Mean cost	Control (N=2,271)	RAPIDO (N=2,197)	Difference (95% CI) [a]
Intervention cost	-	£7	+£7
Antimicrobial cost	£272	£292	+£18 (-£6 to £46)
7-day ward costs	£3,805	£3,757	-£49 (-£182 to £85)
Total 7-day costs	£4,077	£4,055	-£22 (-£163 to £112)
28-day ward costs	£9,325	£9,282	-£43 (-£557 to £471)
Total 28-days costs	£9,597	£9,580	-£17 (-£537 to £503)

Note: *[a] Confidence intervals around mean differences calculated from unadjusted linear regression*

Estimated costs following multiple imputation are presented in *Table 2*.

Table 2 Costs following multiple imputation (based on 30 imputed datasets of N=5,550)

Mean cost	Control (N=2,810)	RAPIDO (N=2,740)	Difference (95% CI) [a]
Total 7-day costs	£3,667	£3,576	-£82 (-£321 to £157)
Total 28-days costs	£8,253	£8,139	-£114 (-£773 to £545)

Note: *[a] Confidence intervals around mean differences calculated from unadjusted linear regression*

Cost-effectiveness and sensitivity analysis

In the base-case imputed analysis, estimated mean costs per patient were lower in the RAPIDO arm (mean difference -£126; 95% CI:-£784 to £532), and the proportion of patients alive at day 28 was also lower (81.4% vs 82.3%, see *Table 3*). Ward costs, including the costs of conventional microbiological testing, constituted 97% of total costs in each arm. Most of the remaining 3% of total cost was attributable to antimicrobial costs. The estimated per-patient cost of diagnosis using MALDI-TOF constituted a negligible proportion of overall per-patient mean costs in the intervention arm.

Table 3 Costs and outcome: base-case analysis with imputation (N=5,550)

	Control	RAPIDO	Difference (95% CI)

For peer review only

Mean 28-day NHS costs	£ 8,259	£8,113	-£126 (-£784 to £532)
28-day survival[a]	0.823	0.814	-0.009 (-0.029 to 0.011)

Note: *[a]Survival measured as the proportion of patients alive at day 28.*

Table 4 Cost-effectiveness: base-case analysis with imputation (N=5,550)

Threshold[a]	Net monetary benefit (95% CI)	Probability of cost-effectiveness
£5,000	£83 (-£567 to £733)	0.59
£10,000	£40 (-£625 to £706)	0.55
£20,000	-£45 (-£783 to £692)	0.45
£30,000	-£131 (-£984 to £721)	0.38
£50,000	-£303 (-£1,460 to £855)	0.30

Note: *[a]Threshold value = 28-day cost per death avoided at 28 days.*

The probability of the RAPIDO intervention being cost-effective declines with increasing threshold values of cost per death avoided at 28 days, as shown in

Figure 1 and *Table 4*.

Figure 1 Cost-effectiveness acceptability curve for base case

Table 5 and *Table 6* report the results of the various sensitivity analyses. These analyses, expressed as net monetary benefit (with associated 95% confidence intervals), do not differ substantially from the base-case results. Estimating costs at seven rather than 28 days did not alter the overall cost-effectiveness conclusions.

Subgroup analysis

Table 7 and *Table 8* present the results of the subgroup analysis comparing clinically significant and clinically non-significant episodes of bloodstream infection. Statistical tests for interaction showed no evidence of a subgroup effect (p-value for interaction in the cost seemingly unrelated regression equation=0.32, p-value in the outcome seemingly unrelated regression equation =0.66), and estimates of difference between Conventional and RAPIDO diagnosis in both outcome and costs were broadly similar for the clinically significant and clinically non-significant subgroups.

For peer review only

For peer review only

Table 5 Sensitivity analysis: costs and outcome

		Excluding unapproached survivors, N=4,468, cost at 28 days		Excluding unapproached survivors, N=4,468, cost at 7 days		Including unapproached survivors, N=5,550, cost at 7 days	
NHS costs mean (95% CI)	Control	£9,604	(£9,243 to £9,967)	£4,079	(£3,982 to £4,117)	£3,669	(£3,500 to £3,836)
	RAPIDO	£9,572	(£9,204 to £9,441)	£4,053	(£3,953 to £4,153)	£3,574	(£3,378 to £3,770)
	Difference	-£33	(-£549 to £484)	-£26	(-£166 to £113)	-£95	(-£358 to £168)
28-day survival mean (95% CI)	Control	0.78	(0.76 to 0.80)	0.78	(0.76 to 0.80)	0.82	(0.81 to 0.84)
	RAPIDO	0.77	(0.75 to 0.79)	0.77	(0.75 to 0.79)	0.81	(.80 to 0.83)
	Difference	-0.01	(-0.04 to 0.01)	-0.01	(-0.04 to -0.01)	-0.01	(-0.03 to 0.01)

Table 6 Sensitivity analysis: cost effectiveness

	Excluding unapproached survivors, N=4,468, cost at 28 days			Excluding unapproached survivors, N=4,468, cost at 7 days			Including unapproached survivors, N=5,550, cost at 7 days		
Threshold[a]	NMB	(95% CI)	PCE[b]	NMB	(95% CI)	PCE	NMB	(95% CI)	PCE
£5,000	-£29	(-£533 to £475)	0.46	-£35	(-£206 to £136)	0.34	£52	(-£222 to £326)	0.65
£10,000	-£91	(-£611 to £430)	0.37	-£97	(-£359 to £165)	0.23	£9	(-£314 to £332)	0.52
£20,000	-£214	(-£841 to £414)	0.25	-£220	(-£706 to £267)	0.19	-£77	(-£551 to £398)	0.38
£30,000	-£337	(-£1,135 to £461)	0.20	-£343	(-£1,067 to £381)	0.18	-£163	(-£817 to £492)	0.31
£50,000	-£583	(-£1799 to -£634)	0.17	-£589	(-£1,796 to £618)	0.17	-£334	(-£1,373 to £705)	0.26

Notes: *[a]Threshold value = cost per death avoided at 28 days; [b]NMB = net monetary benefit [mean (95% confidence interval)]; [c]PCE = probability of cost-effectiveness at given threshold.*

For peer review only

For peer review only - http://bmjopen.bmj.com/site/about/guidelines.xhtml

Table 7 Subgroup analysis: costs and outcome

		All (unapproached survivors excluded) N=4,468		Clinically significant episodes only N=3,010 (67%)		Clinically non-significant episodes only N=1,458 (33%)	
28-day NHS costs mean (95% CI)	Control	£9,604	(£9,243 to £9,967)	£9,456	(£8,991 to £9,922)	£9,451	(£8,786 to £10,116)
	RAPIDO	£9,572	(£9,204 to £9,441)	£9,618	(£9,134 to £10,102)	£9,047	(£8,399 to £9,696)
	Difference	-£33	(-£549 to £484)	£161	(-£469 to £791)	-£404	(-£1,308 to £501)
28-day survival mean (95% CI)	Control	0.78	(0.76 to 0.80)	0.78	(0.76 to 0.79)	0.80	(0.77 to 0.83)
	RAPIDO	0.77	(0.75 to 0.79)	0.77	(0.75 to 0.79)	0.78	(0.75 to 0.81)
	Difference	-0.01	(-0.04 to 0.01)	-0.01	(-0.04 to 0.02)	-0.02	(-0.06 to 0.02)

Table 8 Subgroup analysis: cost effectiveness at 28 days

Threshold value[a]	All (unapproached survivors excluded) N=4,468			Clinically significant episodes only N=3,010 (67%)			Clinically non-significant episodes only N=1,458 (33%)		
	NMB[b]	(95% CI)	PCE[c]	NMB	(95% CI)	PCE	NMB	(95% CI)	PCE
£5,000	-£29	(-£533 to £475)	0.46	-£206	(-£853 to £441)	0.27	£301	(-£629 to £1,231)	0.74
£10,000	-£91	(-£611 to £430)	0.37	-£250	(-£947 to £446)	0.24	£198	(-£803 to £1,199)	0.65
£20,000	-£214	(-£841 to £414)	0.25	-£339	(-£1,206 to £527)	0.22	-£9	(-£1,254 to £1,237)	0.49
£30,000	-£337	(-£1,135 to £461)	0.20	-£428	(-£1,521 to £664)	0.22	-£215	(-£1,785 to £1,355)	0.39
£50,000	-£583	(-£1799 to -£634)	0.17	-£606	(-£2,222 to £1,010)	0.23	-£627	(-£2,949 to £1,694)	0.30

[a]Threshold value = cost per death avoided at 28 days; [b]NMB = net monetary benefit [mean (95% confidence interval)]; [c]PCE = probability of cost-effectiveness at given threshold.

For peer review only

Discussion

Bloodstream infections are significant, prevalent clinical events associated with substantial morbidity, [1] mortality [17] and medical cost.[18] There are an estimated 1.2 million episodes of bloodstream infection and 157,000 associated deaths per year in Europe.[1] Identification of the aetiological agent is a critical step in the treatment of bloodstream infection. We performed a within-trial economic evaluation of MALDI-TOF diagnostic technology for the rapid identification of the causative microbial agent in hospitalised patients with bloodstream infection.

The primary analysis showed that the intervention was not likely to be cost-effective, measured using incremental cost and incremental 28-day survival. However, the differences between arms were modest and associated with considerable uncertainty. It is important therefore to reflect on whether the use of this technology outside trial conditions might alter the conclusions of the within-trial evaluation.

One consideration is that a higher MALDI-TOF throughput of machine-positive blood samples would reduce the overall cost per sample in the intervention arm. A value for the number of samples likely to be encountered in routine use was not included in the unit cost calculation in this study because of the exclusion criteria used in the trial: for example, it considered only samples from patients aged 18 and over. A reduction in the direct cost of MALDI-TOF would lower the intervention cost towards that of conventional diagnosis, but would not change patient outcomes.

Our economic evaluation did not calculate the cost of per-sample of conventional identification separately, since such costs are bundled into the ward stay costs and their inclusion would have amounted to double counting. By contrast, the intervention arm involved the use of MALDI-TOF in addition to conventional diagnosis, and hence the per-sample costs of MALDI-TOF are incremental to costs in the control arm. However, as MALDI-TOF has been increasingly adopted in routine practice, experience shows that it is not, in fact, used as an adjunct to conventional approaches, but largely displaces them. In addition, its widespread adoption for use with

For peer review only - http://bmjopen.bmj.com/site/about/guidelines.xhtml

samples from much more common infections (e.g. urinary tract infections) reduces its per-sample costs overall.

As a rough indication of the possible magnitudes of displacement that could be involved specifically for blood cultures, MALDI-TOF offered a usable identification of some 83% of first-bottle samples in the trial, and few of these samples would merit conventional identification in addition. Of the remaining 17% of samples, it is likely that, after further culture to produce colonial isolates, most would be successfully tested by MALDI-TOF without recourse to conventional biochemical methods.

Other considerations suggest that the incremental cost of MALDI-TOF compared to conventional identification could be smaller in “real-world” contexts than that identified in the RAPIDO trial. Longer term reductions in capital, labour and consumable inputs could not be measured within the period of trial-follow-up and are not reflected in the economic evaluation. For example, the MALDI-TOF process requires less physical space in the laboratory compared to conventional approaches, and a substantial long-term switch to the new technology would reduce the capital costs of microbiology laboratories, as would reductions in the cost of MALDI-TOF machines that may come from wider use and greater market competition. Changes in workflow using MALDI-TOF reduce the time required from laboratory staff to complete an identification, meaning that results can be supplied more quickly to clinical staff on wards significantly faster to clinical staff on wards (a median of 35.6 hours after taking the blood sample using MALDI-TOF compared to 54.5 hours using conventional methods in the RAPIDO trial, $p<0.0001$).

Against this, it is important to note that ward costs accounted for 97% of all secondary care costs, and the absence of a significant effect of MALDI-TOF diagnosis in reducing the length of stay and improving survival is a central conclusion of the trial – and one that merits analysis in future research (discussed below). Without evidence for improvements in these outcomes, reductions in the costs of MALDI-TOF diagnosis are plausible but may not materially alter the conclusions of our analysis.

For peer review only - http://bmjopen.bmj.com/site/about/guidelines.xhtml

Strengths and limitations

Strengths

To our knowledge, this is the first economic evaluation conducted alongside a randomised clinical trial evaluating MALDI-TOF for rapid identification as an addition to conventional microbiological and biochemical methods in bloodstream infection. There has been much observational evidence on the effects of MALDI-TOF technology on non-health outcomes such as time to identification, but there is a lack of trial-based evidence on the costs and cost-effectiveness of using MALDI-TOF in clinical contexts.[19]

The cost-effectiveness analysis was conducted on a large dataset offering individual-level cost data on thousands of patients. The costing of the intervention itself was supported by a time and motion observational study conducted at one of the trial sites, while ward and antimicrobial data were valued using nationally representative data sources.

Limitations

Mortality data was censored at 28 days, and information on ward type (by far the biggest cost driver) at seven days. We lacked the information necessary to examine longer-term costs and outcomes, albeit there is little reason to suspect that survival curves would diverge after 28 days to alter the primary survival outcome of the trial.

The cost analysis depended on a matching of the ward descriptions reported in the trial dataset to 'Service Descriptions' in NHS Reference Costs. This matching process was performed 'blind' to allocation, but the analysis of 28-day costs required an extrapolation from seven days to the earliest of death, discharge or the 28-day end-point. Unobserved changes in ward type after day 7 could change the estimated costs used in the base case, although no substantial difference was observed when comparing 28-day and 7-day analyses.

We did not have access to primary care records so our analysis was limited to a secondary care NHS perspective i.e. hospital resource use. In practice, because of the magnitude of per-day ward costs, it is unlikely that accounting for other health system costs would have a material impact on our findings, given that the mortality outcome favoured conventional diagnosis.

For peer review only - http://bmjopen.bmj.com/site/about/guidelines.xhtml

Patients who were unable to consent for themselves and for whom no consultee was available comprised 19.5% (n=1,082) of the 5,550 patients in the analysis population, and no data beyond the mortality outcome, allocation, age and sex was available for them. However, apart from this group of patients, and the absence of ward-specific data between days 8 and 28, the amount of missing data in key cost drivers was limited.

The multiple imputation model estimated that these 1,082 patients had lower mean costs than the other 4,468 participants. Is this plausible? On the one hand, costs should be expected to diverge between the two groups given that the 1,082 patients who did not provide consent are all known to have survived for at least 28 days. Thus, the lower costs generated by the imputation model may reflect a population less acutely ill than the other participants. On the other hand, prolonged survival without discharge would give rise to higher costs than early death during the trial period.

These considerations complicate assessments of direction of the biases in the available case data. However, the conclusions that emerge from the multiple imputation results, the available case results, and the various sensitivity analyses are similar in identifying considerable uncertainty around the cost-effectiveness of MALDI-TOF in this patient group.

Finally, the economic evaluation was limited to identifying the cost-effectiveness of the intervention and did not identify the mechanisms that gave rise to the survival outcomes in the trial. Evidence from the analysis of secondary clinical outcomes in the RAPIDO trial indicates that time to provision of microbiological identification to the ward was significantly shorter in the RAPIDO arm and there was weak evidence of longer time to initiation of appropriate antimicrobial therapy in the RAPIDO arm (median 24.0 versus 13.0 hours, p=0.056). However, there was no significant difference between arms in other secondary outcomes: time to providing Gram stain and antimicrobial susceptibility results to the ward; time to resolution of fever (up to seven days) or discharge (up to 28 days); *C. difficile* incidence (to 28 days); in-hospital antimicrobial consumption (to seven days) or the proportion of patients remaining on broad-spectrum therapy at seven days [2].

For peer review only - http://bmjopen.bmj.com/site/about/guidelines.xhtml

Future research

Future research could examine the mechanisms by which mortality outcomes may differ between MALDI-TOF and conventional diagnosis. Analysis of length of stay and survival in observational study designs in US contexts by Huang et al[20] and Perez et al [21] found beneficial effects of MALDI-TOF when used in conjunction with antimicrobial stewardship programmes, hinting at organisational changes that may be needed to exploit the faster identification offered by MALDI-TOF [22] . However, this is context-specific, as in other health systems, such as the NHS in which the present trial was conducted, bacteraemia consultation teams are routine and involved in care at an early stage.

Conclusion

Overall, the evidence from the RAPIDO trial suggests that the use of MALDI-TOF as an adjunct to conventional microbial identification is unlikely to offer value when its incremental costs are compared to 28-day incremental survival. It is plausible that the costs of MALDI-TOF in "real-world" routine use may well be lower than those measured during the RAPIDO trial, and savings can also be expected as it would displace much conventional testing.

Author contributions: PD and WH: conducted the economic analysis. PD wrote the first draft of this manuscript. PD, WH, KP, RR, MS, AMG: Reviewed, commented and edited the manuscript. AMG: Chief investigator for the RAPIDO trial.

Data statement: Permission to share individual-level data was not obtained from trial participants. Requests for summary level data should be directed to AMG.

For peer review only - http://bmjopen.bmj.com/site/about/guidelines.xhtml

References

1. Goto M, Al-Hasan MN. Overall burden of bloodstream infection and nosocomial bloodstream infection in North America and Europe. *Clin Microbiol Infect* 2013;19(6):501-09. doi: 10.1111/1469-0691.12195
2. MacGowan A, Grier S, Stoddart M, et al. Impact of rapid microbial identification on clinical outcomes in bloodstream infection: the RAPIDO randomised trial. *Clin Microbiol Infect* 2020 doi: https://doi.org/10.1016/j.cmi.2020.01.030
3. Timbrook TT, Morton JB, McConeghy KW, et al. The Effect of Molecular Rapid Diagnostic Testing on Clinical Outcomes in Bloodstream Infections: A Systematic Review and Meta-analysis. *Clinical infectious diseases : an official publication of the Infectious Diseases Society of America* 2017;64(1):15-23. doi: 10.1093/cid/ciw649 [published Online First: 2016/11/03]
4. Albur MS, Bowker K, Weir I, et al. Factors influencing the clinical outcome of methicillin-resistant Staphylococcus aureus bacteraemia. *European journal of clinical microbiology & infectious diseases : official publication of the European Society of Clinical Microbiology* 2012;31(3):295-301. doi: 10.1007/s10096-011-1310-2 [published Online First: 2011/06/15]
5. Laupland KB, Gregson DB, Church DL, et al. Incidence, risk factors and outcomes of Escherichia coli bloodstream infections in a large Canadian region. *Clinical microbiology and infection : the official publication of the European Society of Clinical Microbiology and Infectious Diseases* 2008;14(11):1041-7. doi: 10.1111/j.1469-0691.2008.02089.x [published Online First: 2008/12/02]
6. van Hal SJ, Jensen SO, Vaska VL, et al. Predictors of mortality in Staphylococcus aureus Bacteremia. *Clinical microbiology reviews* 2012;25(2):362-86. doi: 10.1128/cmr.05022-11 [published Online First: 2012/04/12]
7. Seifert H. The Clinical Importance of Microbiological Findings in the Diagnosis and Management of Bloodstream Infections. *Clin Infect Dis* 2009;48(Supplement 4):S238-S45. doi: http://dx.doi.org/10.1086/598188
8. Evans RN, Pike K, Rogers CA, et al. Modifiable healthcare factors affecting 28-day survival in bloodstream infection: a prospective cohort study. *BMC Infect Dis* 2020;20(1):545. doi: 10.1186/s12879-020-05262-6
9. Fournier P-E, Drancourt M, Colson P, et al. Modern clinical microbiology: new challenges and solutions. *Nature Reviews Microbiology* 2013;11(8):574–85. doi: http://dx.doi.org/10.1038/nrmicro3068
10. Curtis L, Burns A. Unit Costs of Health and Social Care 2019. Personal Social Services Research Unit. University of Kent, Canterbury, 2018.
11. Department of Health. Reference Costs 2012/13. London, 2013.
12. Joint Formulary Committee. British National Formulary (BNF): Pharmaceutical Press, 2012.
13. White IR, Royston P, Wood AM. Multiple imputation using chained equations: Issues and guidance for practice. *Stat Med* 2011;30(4):377-99. doi: 10.1002/sim.4067
14. Royston P, White IR. Multiple Imputation by Chained Equations (MICE): Implementation in Stata. *Journal of Statistical Software* 2011;45:1-20.
15. Faria R, Gomes M, Epstein D, et al. A Guide to Handling Missing Data in Cost-Effectiveness Analysis Conducted Within Randomised Controlled Trials. *Pharmacoeconomics* 2014;32(12):1157-70. doi: 10.1007/s40273-014-0193-3
16. Willan AR, Briggs AH, Hoch JS. Regression methods for covariate adjustment and subgroup analysis for non-censored cost-effectiveness data. *Health Econ* 2004;13(5):461-75. doi: 10.1002/hec.843

For peer review only - http://bmjopen.bmj.com/site/about/guidelines.xhtml

17. Bearman GML, Wenzel RP. Bacteremias: A Leading Cause of Death. *Arch Med Res* 2005;36(6):646-59. doi: http://dx.doi.org/10.1016/j.arcmed.2005.02.005
18. Kilgore M, Brossette S. Cost of bloodstream infections. *Am J Infect Control* 2008;36(10):S172.e1-S72.e3.
19. Dixon P, Davies P, Hollingworth W, et al. A systematic review of matrix-assisted laser desorption/ionisation time-of-flight mass spectrometry compared to routine microbiological methods for the time taken to identify microbial organisms from positive blood cultures. *Eur J Clin Microbiol Infect Dis* 2015;34(5):863-76. doi: 10.1007/s10096-015-2322-0
20. Huang AM, Newton D, Kunapuli A, et al. Impact of Rapid Organism Identification via Matrix-Assisted Laser Desorption/Ionization Time-of-Flight Combined With Antimicrobial Stewardship Team Intervention in Adult Patients With Bacteremia and Candidemia. *Clin Infect Dis* 2013;57(9):1237-45. doi: http://dx.doi.org/10.1093/cid/cit498
21. Perez KK, Olsen RJ, Musick WL, et al. Integrating Rapid Pathogen Identification and Antimicrobial Stewardship Significantly Decreases Hospital Costs. *Arch Pathol Lab Med* 2012;137(9):1247-54. doi: http://dx.doi.org/10.5858/arpa.2012-0651-OA
22. Kothari A, Morgan M, Haake D. Emerging Technologies for Rapid Identification of Bloodstream Pathogens. *Clin Infect Dis* 2014

For peer review only

For peer review only - http://bmjopen.bmj.com/site/about/guidelines.xhtml

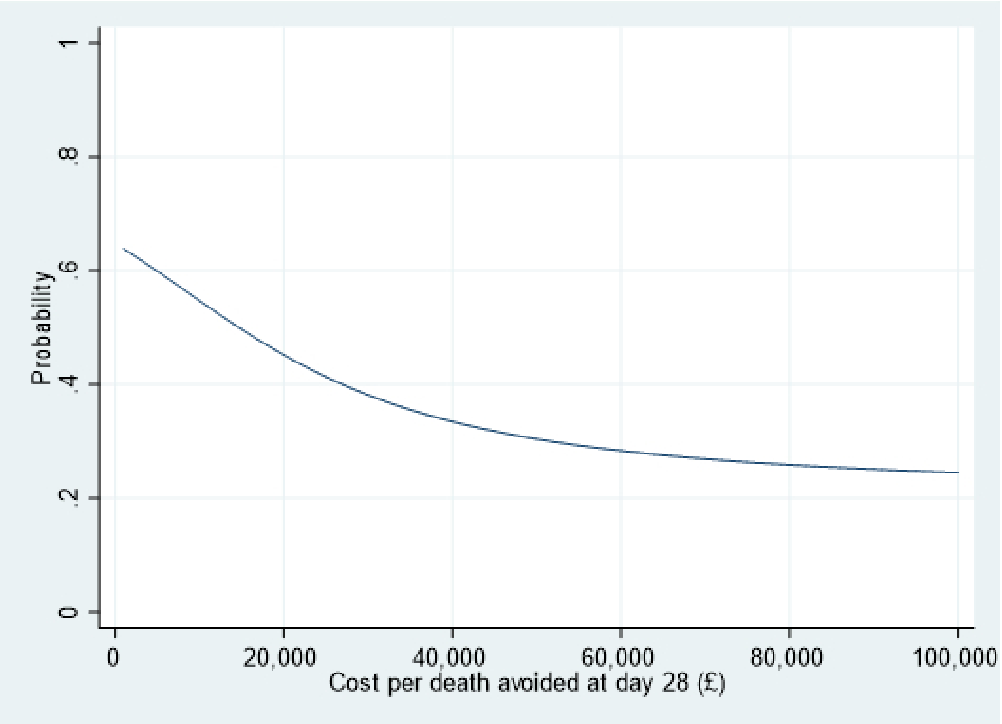

Cost-effectiveness acceptability curve for base case

SUPPLEMENTARY MATERIAL - Cost-effectiveness of rapid laboratory-based mass-spectrometry diagnosis of bloodstream infection: Evidence from the RAPIDO randomized controlled trial

Costing of hospital stays

The cost of ward stays was calculated from the 2012/13 NHS Reference Costs (1) and then inflated using Curtis and Burns (2) as described in main text.

Costing by ward speciality

Hospital admission was an inclusion criterion for the RAPIDO trial, but a small number of "non-admitted" ward-days could occur in cases where the diagnostic blood sample was taken in an outpatient or day-case setting shortly before admission to a more appropriate speciality. Therefore, all ward stays were costed as inpatients unless the ward speciality recorded in the dataset explicitly specified otherwise e.g. day-case surgery.

The ward specialities described in the dataset were mapped to their nearest equivalent Service Descriptions and currency codes in NHS Reference Costs as shown in *Table* . Where more than one Service Description or currency code applied to a ward speciality, the ward cost was calculated as an average of the unit costs of each Service Description or currency code, weighted by the number of bed-days reported for them in NHS Reference Costs. An example calculation is shown in **Error! Reference source not found.**.

The RAPIDO dataset did not include the number of organs supported in critical care so the cost of stays in high-dependency units (HDUs) and intensive therapy units (ITUs) was also calculated as a weighted average. The costs of a bed-day on an adult critical care unit for the different levels of organ support are listed in NHS Reference Costs; these costs were weighted by the number of bed-days reported for patients at each level of support to give an average cost for use in analysis.

For peer review only

Table A1 Matching of RAPIDO ward specialities to NHS Reference Cost categories

**RAPIDO ward speciality description**	**NHS Reference Cost 'Service Description'**	**NHS Reference Cost 'service code' or 'currency description'**[a,b]
Medical: Acute medical admissions and pre-admissions	Accident and Emergency	Admitted codes only[c]
Medical: Cardiology / cardiovascular / coronary	Cardiology	320
Medical: Care of the Elderly	Geriatric Medicine	430
Medical: Dermatology / rheumatology	Dermatology AND Rheumatology	410[R] AND 330[D]
Medical: Diabetes / endocrinology	Diabetic Medicine AND Endocrinology	307[D] AND 302[E]
Medical: Gastroenterology / gastrology / liver	Gastroenterology AND Hepatology	301 [G] and 306[H]
Medical: General medical (no declared speciality)	General Medicine	300
Medical: Haematology / oncology	Clinical haematology AND Clinical oncology	303[H] and 800[O]
Medical: Infectious disease / travel medicine	Infectious Diseases	350
Medical: Nephrology / renal / dialysis	Nephrology	361
Medical: Neurology / neurosciences / neuromedical	Neurology	400
Medical: Palliative	Palliative Medicine	315
Medical: Respiratory	Respiratory Medicine	328
Medical: Stroke	Stroke Medicine	340
ITU/HDU[d]: General (not specified as surgical, medical or specialist)	Adult Critical Care Unit	Critical care currencies
ITU/HDU: General medical	N/A[e]	N/A
ITU/HDU: General surgical	Adult Critical Care Unit	Critical care currencies
ITU/HDU: Cardiac	Adult Critical Care Unit	Critical care currencies
ITU/HDU: Neurology/neurosurgery	Adult Critical Care Unit	Critical care currencies
ITU/HDU: Renal	N/A	N/A
ITU/HDU: Theatre recovery areas	N/A	N/A
Surgery: Admissions / pre-admissions units	General surgery	100
Surgery: Cardiothoracic / thoracic	Cardiothoracic surgery AND Thoracic surgery	170[C] and 173[T]
Surgery: Ear, nose, throat, oral & maxillo-facial, and opthalmic units	All surgical codes[f]	Inpatient codes only[c]
Surgery: General including GI, breast, vascular	General surgery	100

For peer review only

Surgery: Neurosurgery	Neurosurgery	150
Surgery: Orthopaedic / trauma	Trauma & Orthopaedics	110
Surgery: Plastics / burns	Plastic surgery	160
Surgery: Short stay and daycase units	General surgery	100, day cases only
Surgery: Urology / renal	All surgical codes[f]	Inpatient codes only[c]
Other: A&E, emergency assessment, fracture clinics and related units	Accident and Emergency	Admitted codes only[c]
Other: Imaging, diagnostics and telemetry	N/A	N/A
Other: Obstetrics & gynaecology	Obstetrics	501
Other: Psychology / psychiatry / mental health	N/A	N/A
Other: Services - not medical, surgical or HDU/ITU, and not listed elsewhere	"Other Procedures or Health Care Problems"	Inpatient codes only[c]

[a] *If there were separate codes for adults and children, only adult codes were used.*

[b]*If multiple NHS Service Descriptions or Currency Codes applied, costs were weighted by the number of bed-days occupancy for each Description or Code (or, in critical care, number of organs supported) reported in NHS Reference Costs.*

[c] *Only 'Inpatient' or 'Admitted' codes were used in these cases as all RAPIDO patients were admitted before or soon after taking of the diagnostic blood sample.*

[d]*HDU/ITU = critical care specialities (high dependency and intensive therapy units), costs weighted by number of bed-days occupancy for each number of organs supported in critical care.*

[e] *N/A = Not applicable – not costed as no observations in the trial.*

[f]*A weighted average across all admitted surgical codes was used for surgical specialities that were not listed specifically in NHS Reference Costs. Costing by length of stay*

Hospitals in the NHS are paid according to patients' length of stay, with different levels of payment being made according to whether each bed-day of the stay is an 'inlier' bed-day or an excess (or 'outlier') bed-day. These payments constitute 'costs' from an NHS system perspective and we used them to cost hospital stays.

The total costs of the patients' stays in each arm of the trial depend on their whole ward history from the date of admission, not from the date of randomisation - although, clearly, differences due to the RAPIDO intervention could only accrue after randomisation. The total hospital cost could not be calculated because, although the date of admission was known, it was not known for how long patients had been on particular wards before the diagnostic blood samples were taken.

The distinction between an inlier bed-day and an excess bed-day is defined by reference to a trim point, a figure which is intended to capture the upper end of the range of lengths of stay expected for a given currency code. It is calculated as the (upper quartile of length of stay) + 1.5 × (interquartile range of length of stay). In a long stay, days after the trim point are counted as excess bed-days, while days up to the trim point are counted as inliers.

For inlier costs, national average unit costs and average length of stay (number of days) per Finished Consultant Episode are reported. A Finished Consultant Episode is a completed episode of treatment

For peer review only

received by a patient under the care of one consultant. Dividing the mean nationwide unit cost for a given currency code by the mean nationwide number of inpatient days for a patient admitted under that currency code gives its estimated per-day cost. To obtain the weighted cost for each currency code, we multiply the per-day cost by the share of that currency code in the total bed-days of all currency codes in each Service Description. The sum of these figures is the weighted average per-day cost for either elective inpatient or non-elective inpatients receiving treatment under that Service Description. Unit costs per excess bed day are calculated in a similar manner.

Elective and non-elective stays

The RAPIDO dataset did not include information to classify each day of each patient's stay in a ward as elective or non-elective. It is likely that a high proportion of ward admittances after day 0 of the trial were non-elective since they followed a presumed diagnosis (or, at least, clear suspicion) of bloodstream infection, as evidenced by the taking of a blood sample for culture. However, it is possible that the infection developed during an elective admission, so ward costs – particularly on day 0 – might in fact be charged as elective. We therefore included both elective and non-elective costs in our calculations, by weighting the average cost of all currency codes in each category (elective and non-elective) by their respective shares in bed-days.

A post hoc analysis examined the sensitivity of unit costs to the exclusion of elective care. The effect on unit costs of this exclusion was modest. In some cases, unit costs do not distinguish between elective and non-elective (e.g. for critical care) and the exclusion had no effect, and in other cases the effect of excluding elective admissions was to reduce unit costs by approximately 0.1% to 1%. We consider that including elective care in the calculation of unit costs in general is a conservative approach, and one appropriate to our base-case analysis. We therefore did not re-run this analysis using the very slightly lower unit costs that would have arisen under the exclusion of elective care.

Example: calculation of per-day cost for a 'General Medical' ward stay

The steps involved in the calculation of unit costs for the 'General Medicine' Service Description are set out in Table A2.

Table A2 Example calculation of weighted per-day cost associated with the 'General Medicine' Service Description in 2012/13

Item	Quantity or share of total	Calculation
Number of bed-days[a]		
National total bed-days per year: Elective Inpatient excluding short-stay (EI)	96,339	A
National total bed-days per year: Non-Elective Inpatient (NEI)	6,398,877	B
National total bed-days per year: Non-Elective Inpatient Short-stay (NEI-S)	1,545,596	C
National total bed-days per year: Inpatients (EI + NEI + NEI-S)	8,040,812	D=(A+B+C)
Share of bed-days		

For peer review only

% of days that relate to EI	1.20%	E=A/D
% of days that relate to NEI	79.58%	F=B/D
% of days that relate to NEI-S	19.22%	G=C/D
Averaging over inlier/outlier bed-days and currency codes		
Weighted[b] average per-day cost: EI	£456.18	H[b]
Weighted[b] average per-day cost: NEI	£327.48	I[b]
Weighted[b] average per-day cost: NEI-S	£434.19	J[b]
Contributions of elective, non-elective and short stays		
Weighted[c] EI cost per day	£5.47	K=H × E
Weighted[c] NEI cost per day	£260.61	L=F × I
Weighted[c] NEI-S cost per	£83.46	M=G × J
Estimated mean cost per day for RAPIDO analysis		
Cost of a General Medical ward-day for RAPIDO analysis i.e. weighted average of EI, NEI and NEI-S per day costs	£349.53	K+L+M

[a]'Bed-days' here captures both bed days and excess bed days. [b]These figures are calculated as the average of bed-day-weighted sum of costs for inlier bed-days and excess bed-days across all currency codes within the 'General Medicine' service description, weighted by proportion of bed-days from each currency code, as described in the text. [c]These figures are weighted by the proportion of bed-days from each type of inpatient stay (elective, non-elective, and non-elective short-stay).

Costing of MALDI-TOF spectrometry

Data from published literature and confidential information provided by one study centre was used to estimate a mean per blood-sample cost of diagnosis using MALDI-TOF technology at 2012/13 prices and then subsequently inflated.

At this centre, 4,303 machine-positive adult blood samples were recorded during the whole study period, equating to 2,061 per year. In routine use of the technology, all of these would have been tested by MALDI-TOF but fewer were actually tested during the RAPIDO trial. Only 3,153 flagged positive during study hours (=1510/year) and, with 1:1 randomisation, only approximately half of those (755) would have been allocated to MALDI-TOF diagnosis.

We estimated the proportion of total MALDI-TOF cost attributable to use for positive blood cultures as 12% based on two considerations. First, approximately 10-15% of all microbiology laboratory requests for 'culture and sensitivity' related to blood samples. Second, at this centre, organisms from blood accounted for approximately 12% of all organisms identified.

We used these figures to calculate the capital, operating, labour and consumable costs used per positive sample as set out in Table A3. Salary costs and information concerning on costs and overheads were taken from Curtis (2013).(3)

For peer review only

Table A3 Calculation of unit cost of MALDI-TOF identification in 2012/13 prices

Item	Mean value	Comment / data source
Capital-related costs		
Capital cost for new MALDI-TOF machine	£130,000	Published literature, catalogue prices, confidential information from one study centre
Economic life used for depreciation calculations	10 years	Published literature, catalogue prices, confidential information from one study centre
Annual capital charge per blood sample	£0.76	
Maintenance costs		
Annual non-reagent maintenance contract (13% of capital cost)	£16,900	Published literature, confidential information from one study centre
Maintenance cost per blood sample	£0.98	
Consumable costs		
Consumables cost per blood sample, as used in RAPIDO protocol	£0.17	Trial protocol, catalogue data, information from one study centre
Labour costs, including on-costs and overheads		
Band 5 salary	£62,927	Staff grades at one study centre, published NHS payscales, Curtis (2013) for information on oncosts and overhead
Band 6 salary	£76,569	Staff grades at one study centre; published NHS payscales; information on on-costs and overheads from Curtis (2013).(3)
Labour cost per sample	£4.17	...assuming that each grade contributes equally to processing of all blood samples
Total cost		
Total cost per machine-positive blood sample analysed with MALDI-TOF	£6.08	

For peer review only

Costs and results in 2012/13 price levels

Table A4 Costs in available cases

Mean cost	Control (N=2,271)	RAPIDO (N=2,197)	Difference (95% CI) [a]	
Intervention cost	-	£6	+£6	
Antimicrobial cost	£247	£265	+£18	(-£6 to £41)
7-day ward costs	£3,448	£3,404	-£44	(-£165 to £77)
Total 7-day costs	£3,695	£3,675	-£20	(-£148 to £108)
28-day ward costs	£8,451	£8,412	-£39	(-£505 to £427)
Total 28-days costs	£8,698	£8,682	-£15	(-£487 to £456)

[illegible]: *[a] Confidence intervals around mean differences calculated from unadjusted linear regression*

Cost-effectiveness and sensitivity analysis

[illegible]

Table A5 Costs and outcome: base-case analysis with imputation (N=5,550)

	Control	RAPIDO	Difference (95% CI)	
Mean 28-day NHS costs	£ 7,485	£7,371	-£114	(-£710 to £482)
28-day survival[a]	0.823	0.814	-0.009	(-0.029 to 0.011)

[illegible]*[a]Survival measured as the proportion of patients alive at day 28.*

Table A6 Cost-effectiveness: base-case analysis with imputation (N=5,550)

Threshold[a]	Net monetary benefit (95% CI)		Probability of cost-effectiveness
£5,000	£71	(-£519 to £661)	0.59
£10,000	£28	(-£579 to £636)	0.54
£20,000	-£57	(-£745 to £630)	0.44
£30,000	-£143	(-£954 to £668)	0.36
£50,000	-£315	(-£1,443 to £814)	0.29

[illegible]*[a]Threshold value = 28-day cost per death avoided at 28 days.*

[illegible]

For peer review only

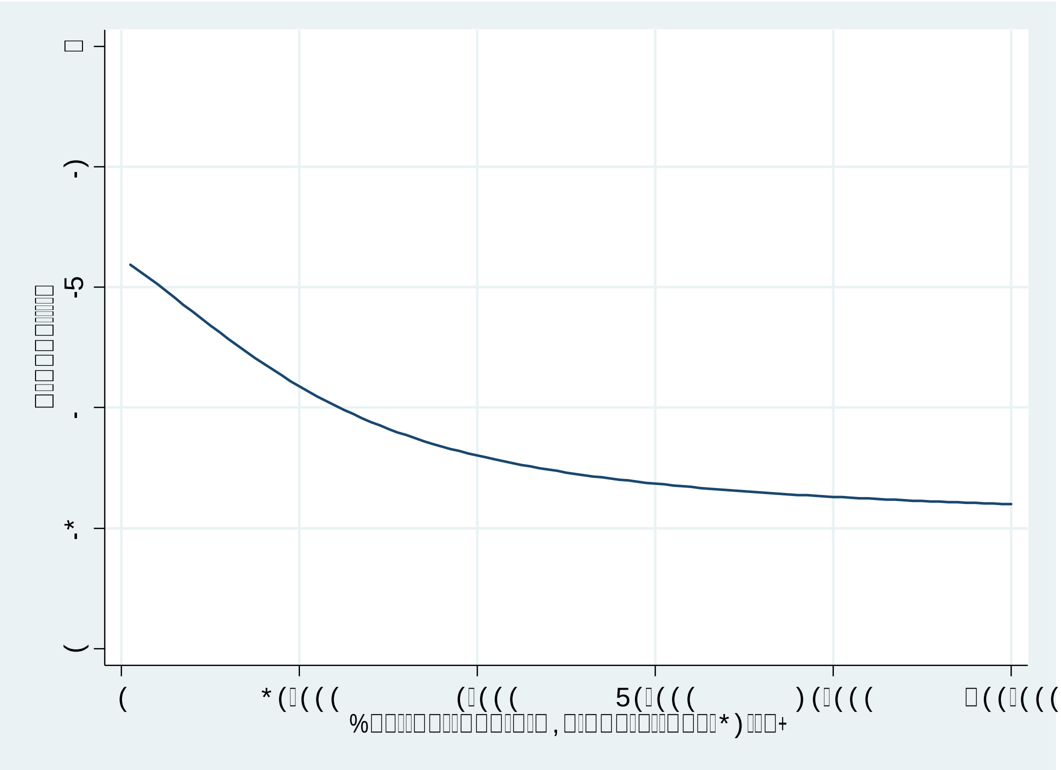

Figure A1 Cost-effectiveness acceptability curve for base case

[illegible]

Subgroup analysis

[illegible]

Table A7 Sensitivity analysis: costs and outcome

		Excluding unapproached survivors, N=4,468, cost at 28 days		Excluding unapproached survivors, N=4,468, cost at 7 days		Including unapproached survivors, N=5,550, cost at 7 days	
NHS costs mean (95% CI)	Control	£8,705	(£8,376 to £9,033)	£3,697	(£3,608 to £3,786)	£3,324	(£3,098 to £3,598)
	RAPIDO	£8,675	(£8,341 to £9,009)	£3,673	(£3,583 to £3,763)	£3,238	(£3,094 to £3,619)
	Difference	-£30	(-£498 to £439)	-£24	(-£151 to £103)	-£86	(-£324 to £153)
28-day survival mean (95% CI)	Control	0.78	(0.76 to 0.80)	0.78	(0.76 to 0.80)	0.82	(0.81 to 0.84)
	RAPIDO	0.77	(0.75 to 0.79)	0.77	(0.75 to 0.79)	0.81	(.80 to 0.83)
	Difference	-0.01	(-0.04 to 0.01)	-0.01	(-0.04 to -0.01)	-0.01	(-0.03 to 0.01)

Table A8 Sensitivity analysis: cost effectiveness

	Excluding unapproached survivors, N=4,468, cost at 28 days			Excluding unapproached survivors, N=4,468, cost at 7 days			Including unapproached survivors, N=5,550, cost at 7 days		
Threshold[a]	NMB	(95% CI)	PCE[b]	NMB	(95% CI)	PCE	NMB	(95% CI)	PCE
£50,000	-£32	(-£489 to £425)	0.45	-£38	(-£199 to £124)	0.32	£43	(-£209 to £295)	0.63
£10,000	-£94	(-£572 to £385)	0.35	-£99	(-£356 to £158)	0.23	£0	(-£305 to -£305)	0.50
£20,000	-£217	(-£814 to £381)	0.24	-£222	(-£707 to £263)	0.18	-£86	(-£548 to £377)	0.36
£30,000	-£340	(-£1,117 to £438)	0.20	-£345	(-£1,069 to £379)	0.17	-£171	(-£817 to £474)	0.30
£50,000	-£586	(-£1,793 to £622)	0.17	-£591	(-£1,799 to £616)	0.17	-£343	(-£1,376 to £691)	0.26

[a]Threshold value = cost per death avoided at 28 days; [b]NMB = net monetary benefit [mean (95% confidence interval)]; [c]PCE = probability of cost-effectiveness at given threshold.

For peer review only

Table A9 Subgroup analysis: costs and outcome

		All (unapproached survivors excluded) N=4,468		Clinically significant episodes only N=3,010 (67%)		Clinically non-significant episodes only N=1,458 (33%)	
28-day NHS costs mean (95% CI)	Control	£8,705	(£8,376 to £9,033)	£8,570	(£8,148 to £8,992)	£8,565	(£7,962 to £9,168)
	RAPIDO	£8,675	(£8,341 to £9,009)	£8,716	(£8,278 to £9,155)	£8,199	(£7,611 to £8,787)
	Difference	-£30	(-£498 to £439)	£146	(-£425 to £717)	-£365	(-£1,186 to £454)
28-day survival mean (95% CI)	Control	0.78	(0.76 to 0.80)	0.78	(0.76 to 0.79)	0.80	(0.77 to 0.83)
	RAPIDO	0.77	(0.75 to 0.79)	0.77	(0.75 to 0.79)	0.78	(0.75 to 0.81)
	Difference	-0.01	(-0.04 to 0.01)	-0.01	(-0.04 to 0.02)	-0.02	(-0.06 to 0.02)

Table A10 Subgroup analysis: cost effectiveness at 28 days

Threshold value[a]	All (unapproached survivors excluded) N=4,468			Clinically significant episodes only N=3,010 (67%)			Clinically non-significant episodes only N=1,458 (33%)		
	NMB[b]	(95% CI)	PCE[c]	NMB	(95% CI)	PCE	NMB	(95% CI)	PCE
£50,000	-£32	(-£489 to £425)	0.45	-£191	(-£780 to £390)	0.26	£263	(-£585 to £1,110)	0.73
£10,000	-£94	(-£572 to £385)	0.35	-£235	(-£879 to £409)	0.24	£160	(£765 to £1,085)	0.63
£20,000	-£217	(-£814 to £381)	0.24	-£324	(-£1,149 to £501)	0.22	-£47	(-£1,231 to £1,138)	0.47
£30,000	-£340	(-£1,117 to £438)	0.20	-£413	(-£1,472 to £647)	0.22	-£253	(-£276 to -£230)	0.37
£50,000	-£586	(-£1,793 to £622)	0.17	-£591	(-£2,814 to £1,003)	0.23	-£665	(-£700 to -£631)	0.28

[a]Threshold value = cost per death avoided at 28 days; [b]NMB = net monetary benefit [mean (95% confidence interval)]; [c]PCE = probability of cost-effectiveness at given threshold.

References

For peer review only

1. **Department of Health** (2013) Reference Costs 2012/13. London.

2. **Curtis L & Burns A** (2018) Unit Costs of Health and Social Care 2019. Personal Social Services Research Unit. University of Kent, Canterbury.

3. **Curtis L** (2013) *Unit Costs of Health and Social Care 2013*. Canterbury: Personal Social Services Research Unit.

For peer review only

CHEERS checklist—Items to include when reporting economic evaluations of health interventions

Section/item	Item No	Recommendation	Reported on page No/ line No
**Title and abstract**			
Title	1	Identify the study as an economic evaluation or use more specific terms such as "cost-effectiveness analysis", and describe the interventions compared.	P1
Abstract	2	Provide a structured summary of objectives, perspective, setting, methods (including study design and inputs), results (including base case and uncertainty analyses), and conclusions.	P1
**Introduction**			
Background and objectives	3	Provide an explicit statement of the broader context for the study.	P5-6
		Present the study question and its relevance for health policy or practice decisions.	P5-6
**Methods**			
Target population and subgroups	4	Describe characteristics of the base case population and subgroups analysed, including why they were chosen.	P5-6
Setting and location	5	State relevant aspects of the system(s) in which the decision(s) need(s) to be made.	P5-6
Study perspective	6	Describe the perspective of the study and relate this to the costs being evaluated.	P5
Comparators	7	Describe the interventions or strategies being compared and state why they were chosen.	P5
Time horizon	8	State the time horizon(s) over which costs and consequences are being evaluated and say why appropriate.	P7
Discount rate	9	Report the choice of discount rate(s) used for costs and outcomes and say why appropriate.	N/A
Choice of health outcomes	10	Describe what outcomes were used as the measure(s) of benefit in the evaluation and their relevance for the type of analysis performed.	N/A

For peer review only

Section/item	Item No	Recommendation	Reported on page No/ line No
Measurement of effectiveness	11a	*Single study-based estimates:* Describe fully the design features of the single effectiveness study and why the single study was a sufficient source of clinical effectiveness data.	P25-7
	11b	*Synthesis-based estimates*: Describe fully the methods used for identification of included studies and synthesis of clinical effectiveness data.	N/A
Measurement and valuation of preference based outcomes	12	If applicable, describe the population and methods used to elicit preferences for outcomes.	N/A
Estimating resources and costs	13a	*Single study-based economic evaluation:* Describe approaches used to estimate resource use associated with the alternative interventions. Describe primary or secondary research methods for valuing each resource item in terms of its unit cost. Describe any adjustments made to approximate to opportunity costs.	P7-9
	13b	*Model-based economic evaluation:* Describe approaches and data sources used to estimate resource use associated with model health states. Describe primary or secondary research methods for valuing each resource item in terms of its unit cost. Describe any adjustments made to approximate to opportunity costs.	N/A
Currency, price date, and conversion	14	Report the dates of the estimated resource quantities and unit costs. Describe methods for adjusting estimated unit costs to the year of reported costs if necessary. Describe methods for converting costs into a common currency base and the exchange rate.	P7-9
Choice of model	15	Describe and give reasons for the specific type of decision-analytical model used. Providing a figure to show model structure is strongly recommended.	N/A

For peer review only

Section/item	Item No	Recommendation	Reported on page No/ line No
Assumptions	16	Describe all structural or other assumptions underpinning the decision-analytical model.	N/A
Analytical methods	17	Describe all analytical methods supporting the evaluation. This could include methods for dealing with skewed, missing, or censored data; extrapolation methods; methods for pooling data; approaches to validate or make adjustments (such as half cycle corrections) to a model; and methods for handling population heterogeneity and uncertainty.	P9-11
**Results**			
Study parameters	18	Report the values, ranges, references, and, if used, probability distributions for all parameters. Report reasons or sources for distributions used to represent uncertainty where appropriate. Providing a table to show the input values is strongly recommended.	P11-12
Incremental costs and outcomes	19	For each intervention, report mean values for the main categories of estimated costs and outcomes of interest, as well as mean differences between the comparator groups. If applicable, report incremental cost-effectiveness ratios.	P11-12
Characterising uncertainty	20a	*Single study-based economic evaluation:* :Describe the effects of sampling uncertainty for the estimated incremental cost and incremental effectiveness parameters, together with the impact of methodological assumptions (such as discount rate, study perspective).	P12-16
	20b	*Model-based economic evaluation:* Describe the effects on the results of uncertainty for all input parameters, and uncertainty related to the structure of the model and assumptions.	N/A
Characterising heterogeneity	21	If applicable, report differences in costs, outcomes, or cost-effectiveness that can be explained by variations between subgroups of patients with different baseline characteristics or	N/A

For peer review only

Section/item	Item No	Recommendation	Reported on page No/ line No
		other observed variability in effects that are not reducible by more information.	
**Discussion**			
Study findings, limitations, generalisability, and current knowledge	22	Summarise key study findings and describe how they support the conclusions reached. Discuss limitations and the generalisability of the findings and how the findings fit with current knowledge.	P17-21
**Other**			
Source of funding	23	Describe how the study was funded and the role of the funder in the identification, design, conduct, and reporting of the analysis. Describe other non-monetary sources of support.	P1
Conflicts of interest	24	Describe any potential for conflict of interest of study contributors in accordance with journal policy. In the absence of a journal policy, we recommend authors comply with International Committee of Medical Journal Editors recommendations.	P2

For consistency, the CHEERS statement checklist format is based on the format of the CONSORT statement checklist

For peer review only

BMJ Open

Cost-effectiveness of rapid laboratory-based mass-spectrometry diagnosis of bloodstream infection: Evidence from the RAPIDO randomized controlled trial

Journal:	*BMJ Open*
Manuscript ID	bmjopen-2020-044623.R1
Article Type:	Original research
Date Submitted by the Author:	03-Sep-2021
Complete List of Authors:	Dixon, Padraig; Oxford University,<br>Hollingworth, William; University of Bristol, School of Social and Community Medicine<br>Pike, Katie; Bristol University<br>Reynolds, Rosy; Bristol Medical School<br>Stoddart, Margaret; Southmead Hospital<br>MacGowan, Alasdair; Southmead Hospital
**Primary Subject Heading**:	Health economics
Secondary Subject Heading:	Infectious diseases
Keywords:	HEALTH ECONOMICS, Diagnostic microbiology < INFECTIOUS DISEASES, Infection control < INFECTIOUS DISEASES

SCHOLARONE™ Manuscripts

I, the Submitting Author has the right to grant and does grant on behalf of all authors of the Work (as defined in the below author licence), an exclusive licence and/or a non-exclusive licence for contributions from authors who are: i) UK Crown employees; ii) where BMJ has agreed a CC-BY licence shall apply, and/or iii) in accordance with the terms applicable for US Federal Government officers or employees acting as part of their official duties; on a worldwide, perpetual, irrevocable, royalty-free basis to BMJ Publishing Group Ltd ("BMJ") its licensees and where the relevant Journal is co-owned by BMJ to the co-owners of the Journal, to publish the Work in this journal and any other BMJ products and to exploit all rights, as set out in our licence.

The Submitting Author accepts and understands that any supply made under these terms is made by BMJ to the Submitting Author unless you are acting as an employee on behalf of your employer or a postgraduate student of an affiliated institution which is paying any applicable article publishing charge ("APC") for Open Access articles. Where the Submitting Author wishes to make the Work available on an Open Access basis (and intends to pay the relevant APC), the terms of reuse of such Open Access shall be governed by a Creative Commons licence – details of these licences and which Creative Commons licence will apply to this Work are set out in our licence referred to above.

Other than as permitted in any relevant BMJ Author's Self Archiving Policies, I confirm this Work has not been accepted for publication elsewhere, is not being considered for publication elsewhere and does not duplicate material already published. I confirm all authors consent to publication of this Work and authorise the granting of this licence.

For peer review only

Cost-effectiveness of rapid laboratory-based mass-spectrometry diagnosis of bloodstream infection: Evidence from the RAPIDO randomized controlled trial

Padraig Dixon[1*], William Hollingworth[2], Katie Pike[3], Rosy Reynolds[2], Margaret Stoddart[4], Alasdair MacGowan[4,5]

1 Nuffield Department of Primary Care Health Sciences, University of Oxford

2 Population Health Sciences, University of Bristol

3 Bristol Trials Centre (Clinical Trials and Evaluation Unit), University of Bristol

4 Infection Sciences, North Bristol NHS Trust, Bristol

5 School of Cellular and Molecular Medicine, University of Bristol

*Corresponding author

Email: Padraig.Dixon@phc.ox.ac.uk

Short title: Cost-effectiveness of rapid bacteraemia identification

Key words: Bloodstream infection, randomised controlled trial, MALDI-TOF, mass spectrometry, cost-effectiveness.

Word count: 3,953

For peer review only

Abstract

Objectives and intervention

Bloodstream infection, the presence of viable microorganisms in the blood, is a prevalent clinical event associated with substantial mortality. Patient outcomes may be improved when the causative microorganism is identified quickly. We assessed the cost-effectiveness of rapid microbial identification by matrix-assisted laser desorption/ionisation time-of-flight (MALDI-TOF) mass spectrometry.

Design

Economic evaluation alongside a randomised multicentre trial (RAPIDO: RAPId Diagnosis on Outcome) assessing the impact of rapid identification by MALDI-TOF spectrometry.

Setting

Adult in-patients with bloodstream infections at seven NHS hospital trusts in England and Wales.

Primary outcome

Net monetary benefit, estimated as incremental costs compared with incremental 28-day survival, of rapid identification by MALDI-TOF spectrometry compared to conventional identification.

Methods

Patients were randomised (1:1) to receive diagnosis by conventional methods of microbial identification (conventional arm) only or by MALDI-TOF spectrometry in addition to conventional identification (RAPIDO arm).

Results

Data from 5,550 patients were included in primary analysis. Mean imputed costs in 2018/19 prices per patient were lower by £126 in the RAPIDO arm (95% CI: -£784 to £532) but the proportion of patients alive at day 28 was lower (81.5% versus 82.3%). The probability of cost-

For peer review only

effectiveness of MALDI-TOF was <0.5 at cost-effectiveness thresholds between £20,000 and £50,000.

Conclusions

Adjunctive MALDI-TOF diagnosis was unlikely to be cost-effective when measured as cost per death avoided at 28 days. However, the differences between arms in cost and effect were modest, associated with uncertainty, and may not accurately reflect “real-world” routine use of MALDI-TOF technology in this patient group.

Trial registration/reference: ISRCTN 97107018 / UKCRN 11978

Strengths and limitations of this study

We report an economic evaluation of the first randomized controlled trial of adjunctive matrix-assisted laser desorption/ionisation time of flight (MALDI-TOF) mass spectrometry identification of the causative microorganism in bloodstream infection.

Data on 5,550 patients from the RAPIDO (RAPId Diagnosis on Outcome) trial were used to estimate the cost-effectiveness of MALDI-TOF in comparison to conventional microbiological methods.

Patients were randomized on a 1:1 basis to conventional or adjunctive identification once a positive blood culture was obtained.

We estimated the net monetary benefit, calculated as incremental costs compared with incremental 28-day survival, of rapid identification by adjunctive MALDI-TOF spectrometry compared to conventional identification.

We calculated the cost-effectiveness of the intervention in subgroups defined by the clinical significance of the infection

For peer review only

Background

Bloodstream infection refers to the presence, confirmed by laboratory testing, of viable microorganisms in the bloodstream. These infections are both prevalent and clinically significant. There are estimated to be approximately 1.2 million annual episodes of bloodstream infection in Europe, 500,000 in North America [1], and100,000 in England and Wales [2]. Estimates of overall mortality range from 15-25% at 30 days post-infection to almost 50% at 12-36 months after infection [3-6].

Rapid identification of the causative microbial pathogen may be associated with improved patient outcomes [7 8]. The RAPIDO trial assessed the impact of laboratory-based RAPId Diagnosis on Outcome of bloodstream infections in hospitalised adult patients at seven NHS Hospital Trusts in England and Wales [2]. Rapid diagnosis was by matrix-assisted laser desorption/ionisation time-of-flight (MALDI-TOF) mass spectrometry applied to machine-positive blood cultures. Here we report the results of an economic evaluation of this trial, from an NHS perspective. Its purpose was to estimate the cost-effectiveness of using MALDI-TOF technology in addition to conventional microbiological techniques compared to conventional approaches alone.

Methods

Trial methods were described in MacGowan et al [2] and are summarised here.

Design

RAPIDO was a multicentre prospective randomised (1:1) non-blinded parallel-group trial comparing two approaches to identification of the causative microorganism(s) of bloodstream infection in hospitalised adult patients at seven centres in England and Wales. The primary outcome was 28-day survival, and the two approaches were MALDI-TOF spectrometry in addition to conventional microbiological culture (‘RAPIDO’ arm) or conventional culture only (‘Conventional’ arm).

Setting and participants

Adult patients aged ≥18 years, admitted to hospital for NHS care, and with a blood sample culture-positive for bacteria or fungi were potentially eligible for inclusion, whether or not the

For peer review only - http://bmjopen.bmj.com/site/about/guidelines.xhtml

organisms were considered clinically significant. Patients were not eligible if they were on an end-of-life pathway, had been previously randomised in the study, were prisoners or young offenders in the custody of the prison service, if the attending physician deemed them unsuitable, or if the positive blood culture entered the diagnostic pathway ‘out of hours’ when both MALDI-TOF and conventional identification methods were not equally available for use. Patients were randomised on a 1:1 basis to either the Conventional or RAPIDO arm.

In order to initiate rapid diagnosis quickly, the study design required prompt randomisation when blood cultures flagged positive, so it was necessary to seek consent retrospectively. Research nurses approached patients for consent when they were sufficiently recovered and had capacity, in the opinion of both the clinical team and the research nurse. If a patient did not have capacity, but was thought likely to regain it, then nurses waited for capacity to return. Otherwise, a relative or close friend of the patient was approached as consultee, if available. If patients with capacity were discharged to independent living before consent could be obtained, consent was sought by post.

Patient and public involvement

The public and patient panel involvement group for microbiology at North Bristol NHS Trust was consulted on study design and the material given to patients.

Intervention

Samples in both arms of the trial were tested by the conventional methods in routine use at the microbiology laboratory of the centre concerned, starting with incubation in a blood culture machine. Each laboratory’s standard operating procedures defined the choice of appropriate biochemical tests and antimicrobial panels, depending on all the information about the organism available at the time.

In the RAPIDO arm, samples were first also tested by MALDI-TOF mass spectrometry, which is a relatively new technology for the identification of microbial organisms.[9] Identification may be achieved within minutes, a much shorter time than for conventional identification.[9] Microbial material was tested on Bruker Microflex MALDI-TOF mass spectrometers running Realtime

For peer review only

Classification software version 3.1 with database version 3 (version 4 from February 2014; Bruker Daltonik GmbH, Bremen, Germany).

Data collection and outcomes

Research nurses and laboratory staff collected data on paper data capture forms for later entry to a web-based database. Detailed data collection lasted from day 0 to day 7 after blood sampling, or discharge or death if earlier, and continued until day 28 for the key outcomes of death, discharge and *C. difficile* infection, and for laboratory data if necessary.

Key clinical data relevant to the economic evaluation included dates of admission, blood sampling (date 0), discharge and death, allowing calculation of duration of stay both before and up to 28 days after the onset of bloodstream infection. All relevant antimicrobial prescriptions were recorded from day 0 to day 7 including drug names, doses, routes and frequencies of administration, and the number of doses actually taken on each day. Ward speciality was recorded for the ward where the patient spent most of each day up to day 7.

For the economic analysis, the trial's survival outcome was expressed as the proportion of patients alive at 28 days, so that the cost-effectiveness results could be interpreted as the incremental cost per percent change in the proportion of patients alive at 28 days.

Measurement and valuation of resource use

The economic analysis took a health system (i.e. NHS) secondary-care perspective for costs. The time horizon for the economic analysis was up to 28 days. Costs and outcomes were therefore not discounted. Costs were first calculated in 2012/13 prices to reflect the structure of relative costs within the NHS during the first year of participant recruitment into the RAPIDO RCT. These costs were then inflated to 2018/19 levels. Costs from 2012/13 to 2015/2016 were inflated using the hospital and community health services (HCHS) index [10]. This index was replaced in 2016 by the NHS cost inflation index (NHSCII) which was used to inflate prices from 2015/16 to the 2018/19 prices. Supplementary material contains results for the uninflated 2012/13 price levels.

For peer review only

The measured components of NHS costs in each arm were: diagnostic testing (reflecting differences in the technology randomly allocated for sample diagnosis); length of stay for initial admission and ward type (reflecting different levels of intensity of clinical support); and antimicrobials prescribed. Further details are available in the supplementary material.

Diagnostic testing

Patients in both arms of the study had conventional diagnostic blood testing. The costs of the conventional approach to diagnosis are 'bundled' within NHS reference cost and tariffs categories for hospital admissions. To avoid double counting of these costs, the costs of conventional diagnosis were not separately calculated. The unit cost of identifying an organism directly from a machine-positive blood sample using MALDI-TOF was calculated using a microcosting exercise, described further in the supplementary material.

Length of stay and ward type

Data on the speciality of the ward on which patients were located was recorded up to the seventh day after the positive blood sample was taken. Unit costs by ward type are not provided in national data sources such as NHS Reference Costs.[11] The closest analogue in NHS Reference Costs is that of 'Service Description', which groups together related procedures. The coded ward specialities were therefore matched to the closest category of 'Service Description' contained in NHS Reference Costs.

For each Service Description, we calculated a per-day cost as the average of the costs for relevant currency codes (which combine patients with similar cost implications) weighted by the frequency of codes as reported in NHS Reference Costs. We also accounted for the remuneration of hospitals according to the length of stay of patients, and for differences in elective and non-elective care. Further details are provided in the supplementary information section.

We estimated ward costs between day seven (the last day at which ward type was recorded) and day 28 (the point at which the primary outcome of the RCT was measured) by a simple extrapolation. This involved assuming that, for those patients known to survive to at least day 7,

For peer review only

that the day 7 ward type was the ward type on which patients were located until the earliest of discharge, death or day 28. We assess the sensitivity of the results to this assumption by comparing the primary outcome to costs at 7 days.

Antimicrobial use

Antimicrobial drugs administered were costed to 2012/13 prices using the British National Formulary [12] and then inflated as with other costs to 2018/19. A per-patient, per-day antimicrobial cost was calculated from the recorded prescriptions and number of doses taken each day.

Analytical methods

We adopted an intention-to-treat approach to analysis, in which all costs and outcomes were analysed according to the diagnostic pathway to which participants' samples were randomised, rather than the pathway actually followed. All analysis was conducted using Stata version 15.1 (StataCorp, College Station, Texas, USA).

Imputation of missing data

The amount of missing data was modest for patients who provided consent. Mortality data at 28 days was available for all but two patients. Information on allocation was complete. Data necessary to cost ward stays was incomplete in 4% of cases, and in 12% of cases for antimicrobials. Logistic regression analysis confirmed that for each cost category this missingness was unrelated to randomised allocation; odds ratios (95% confidence intervals) for missing ward and antimicrobial data were 1.09 (0.80 to 1.49) and 0.93 (0.79 to 1.09), respectively. We used mean imputation, stratified by allocation, in order to include these data points in the complete case analysis.

Our base-case analysis used multiple imputation. Of the 5,550 patients in the analysis population, 19.5% (n=1,082) were eligible but unapproached survivors. Only very limited baseline information was available for these patients, in accordance with the ethical approvals received, but their mortality outcome was known. Our base-case analysis used multiple imputation to estimate the 28-day and 7-day costs of the 1,082 unapproached survivors.

For peer review only

Multiple imputation by chained equations was implemented in Stata 15.1 using the –ice – command.[13 14] The imputation model was stratified by trial arm and included all variables for which there was complete information on the 5,550 analysis sample patients (centre, sex, age, and consent status) and total cost at 7 and 28 days for available cases. We also included the 28-day mortality outcome. We assumed that the two patients for whom the mortality outcome was censored before day 28 had died by day 28. Predictive mean matching[13] was used to allow for non-normal distributions of the cost variables. Costs were imputed at the level of 7- and 28-day costs, rather than for the underlying disaggregated components of these costs. The number of imputations (n=30) was selected to be larger than 100 times the proportion of missing data.[13] We reflected variation within and between the imputed datasets in the analysis using the methods described by Faria et al.[15]

Cost-effectiveness analysis

Cost-effectiveness analyses were conducted using seemingly unrelated regressions, in which the outcomes of NHS costs and the proportion of patients alive at 28 days were calculated jointly. We obtained estimates of the mean difference between arms and their standard errors from these regressions, which we used in calculation of the net monetary benefit of the intervention compared to conventional identification. "In the absence of a survival or mortality-specific threshold, we estimated net monetary benefit (NMB) at a range of threshold values (£5,000, £10,000, £20,000, £30,000 and £50,000 per death avoided at 28 days). To quantify uncertainty, we calculated confidence intervals around point estimates of net benefit, and constructed cost-effectiveness acceptability curves.

We undertook a number of sensitivity analyses. We calculated net benefit excluding the group of eligible but unapproached survivor patients. We also calculated cost-effectiveness at seven days (rather than 28) with and without this group of patients in order to assess whether our findings in the base-case were substantially affected by our extrapolation of ward costs beyond day 7 for participants surviving to this point.

For peer review only - http://bmjopen.bmj.com/site/about/guidelines.xhtml

Subgroup analysis

We undertook one pre-specified subgroup analysis to examine the clinical significance of the infection episode. A positive blood culture may reflect the presence of pathogenic organisms multiplying in the patient's bloodstream (clinically significant infection), or an incidental contamination of the blood sample during blood sampling or laboratory processing (not clinically significant). One may imagine relatively higher near-term costs and potentially worse survival outcomes if the infection is clinically significant. The subgroup analysis offers a test of this hypothesis.

We followed the regression-based methods for subgroup analysis set out by Willan et al.[16] by introducing a factorial subgroup/allocation interaction into the cost and effect equations. In addition to calculating the probability of intervention cost-effectiveness for each subgroup, we also inspected the p-value associated with the interaction term in each regression for evidence of effect modification by subgroup. This analysis necessarily excluded the unapproached survivor group for whom information on clinical significance was unavailable.

For peer review only

Results

A total of 14,298 samples were presented for screening as first machine-positive samples from adult patients during the study period. From this total, 5,670 samples were excluded as occurring out-of-hours and the remaining 8,628 samples were randomised to either RAPIDO (n=4,312) or Conventional identification (n=4,316). Excluding those who were ineligible or declined consent resulted in an analysis population of 5,550 patients (2,740 RAPIDO, 2,810 Conventional). An unexpectedly large group of patients survived to at least day 28 but were not approached for consent because they lacked capacity and no suitable consultee could be found for them. The analysis population of 5,550 included 1,082 eligible but unapproached survivors (543 RAPIDO, 539 Conventional).

Outcomes

A slightly higher proportion of patients died by 28 days in the RAPIDO group (18.5% or 508/2,470) than in the Conventional group (17.7% or 497/2,810). The hazard ratio (calculated from Cox proportional hazards regression) was 1.05 (95% CI: 0.93 to 1.19, p=0.42). Median

time to discharge (up to 28 days) was 15 days in both arms (hazard ratio=0.98; 95% CI: 0.90 to 1.06).

There was limited deviation from the protocol with respect to diagnostic pathway: the correct diagnostic pathway was not followed for 6.1% of patients in the intervention arm (133/2196) and 2.1% in the control arm (48/2271).

Costs

Costs from available cases, after mean imputation but before multiple imputation, are presented in *Table 1*. Costs are similar between arms, with the intervention having slightly lower total costs (the sum of intervention, antimicrobial and ward costs) at 7 and 28 days.

Table 1 Costs in available cases

Mean cost	Control (N=2,271)	RAPIDO (N=2,197)	Difference (95% CI) [a]	
Intervention cost	-	£7	+£7	
Antimicrobial cost	£272	£292	+£18	(-£6 to £46)
7-day ward costs	£3,805	£3,757	-£49	(-£182 to £85)
Total 7-day costs	£4,077	£4,055	-£22	(-£163 to £112)
28-day ward costs	£9,325	£9,282	-£43	(-£557 to £471)
Total 28-days costs	£9,597	£9,580	-£17	(-£537 to £503)

Note: *[a] Confidence intervals around mean differences calculated from unadjusted linear regression*

Estimated costs following multiple imputation are presented in *Table 2*.

Table 2 Costs following multiple imputation (based on 30 imputed datasets of N=5,550)

Mean cost	Control (N=2,810)	RAPIDO (N=2,740)	Difference (95% CI) [a]	
Total 7-day costs	£3,667	£3,576	-£82	(-£321 to £157)
Total 28-days costs	£8,253	£8,139	-£114	(-£773 to £545)

Note: *[a] Confidence intervals around mean differences calculated from unadjusted linear regression*

Cost-effectiveness and sensitivity analysis

In the base-case imputed analysis, estimated mean costs per patient were lower in the RAPIDO arm (mean difference -£126; 95% CI:-£784 to £532), and the proportion of patients alive at day 28 was also lower (81.4% vs 82.3%, see *Table 3*). Ward costs, including the costs of

For peer review only - http://bmjopen.bmj.com/site/about/guidelines.xhtml

conventional microbiological testing, constituted 97% of total costs in each arm. Most of the remaining 3% of total cost was attributable to antimicrobial costs. The estimated per-patient cost of diagnosis using MALDI-TOF constituted a negligible proportion of overall per-patient mean costs in the intervention arm.

Table 3 Costs and outcome: base-case analysis with imputation (N=5,550)

	Control	RAPIDO	Difference (95% CI)
Mean 28-day NHS costs	£ 8,259	£8,113	-£126 (-£784 to £532)
28-day survival[a]	0.823	0.814	-0.009 (-0.029 to 0.011)

Note: *[a]Survival measured as the proportion of patients alive at day 28.*

Table 4 Cost-effectiveness: base-case analysis with imputation (N=5,550)

Threshold[a]	Net monetary benefit (95% CI)	Probability of cost-effectiveness
£5,000	£83 (-£567 to £733)	0.59
£10,000	£40 (-£625 to £706)	0.55
£20,000	-£45 (-£783 to £692)	0.45
£30,000	-£131 (-£984 to £721)	0.38
£50,000	-£303 (-£1,460 to £855)	0.30

Note: *[a]Threshold value = 28-day cost per death avoided at 28 days.*

The probability of the RAPIDO intervention being cost-effective declines with increasing threshold values of cost per death avoided at 28 days, as shown in

Figure 1 and *Table 4*.

Figure 1 Cost-effectiveness acceptability curve for base case

Table 5 and *Table 6* report the results of the various sensitivity analyses. These analyses, expressed as net monetary benefit (with associated 95% confidence intervals), do not differ substantially from the base-case results. Estimating costs at seven rather than 28 days did not alter the overall cost-effectiveness conclusions.

Subgroup analysis

Table 7 and *Table 8* present the results of the subgroup analysis comparing clinically significant and clinically non-significant episodes of bloodstream infection. Statistical tests for interaction showed no evidence of a subgroup effect (p-value for interaction in the cost seemingly unrelated

For peer review only

regression equation=0.32, p-value in the outcome seemingly unrelated regression equation =0.66), and estimates of difference between Conventional and RAPIDO diagnosis in both outcome and costs were broadly similar for the clinically significant and clinically non-significant subgroups.

For peer review only

Table 5 Sensitivity analysis: costs and outcome

		Excluding unapproached survivors, N=4,468, cost at 28 days		Excluding unapproached survivors, N=4,468, cost at 7 days		Including unapproached survivors, N=5,550, cost at 7 days	
NHS costs mean (95% CI)	Control	£9,604	(£9,243 to £9,967)	£4,079	(£3,982 to £4,117)	£3,669	(£3,500 to £3,836)
	RAPIDO	£9,572	(£9,204 to £9,441)	£4,053	(£3,953 to £4,153)	£3,574	(£3,378 to £3,770)
	Difference	-£33	(-£549 to £484)	-£26	(-£166 to £113)	-£95	(-£358 to £168)
28-day survival mean (95% CI)	Control	0.78	(0.76 to 0.80)	0.78	(0.76 to 0.80)	0.82	(0.81 to 0.84)
	RAPIDO	0.77	(0.75 to 0.79)	0.77	(0.75 to 0.79)	0.81	(.80 to 0.83)
	Difference	-0.01	(-0.04 to 0.01)	-0.01	(-0.04 to -0.01)	-0.01	(-0.03 to 0.01)

Table 6 Sensitivity analysis: cost effectiveness

	Excluding unapproached survivors, N=4,468, cost at 28 days			Excluding unapproached survivors, N=4,468, cost at 7 days			Including unapproached survivors, N=5,550, cost at 7 days		
Threshold[a]	NMB	(95% CI)	PCE[b]	NMB	(95% CI)	PCE	NMB	(95% CI)	PCE
£5,000	-£29	(-£533 to £475)	0.46	-£35	(-£206 to £136)	0.34	£52	(-£222 to £326)	0.65
£10,000	-£91	(-£611 to £430)	0.37	-£97	(-£359 to £165)	0.23	£9	(-£314 to £332)	0.52
£20,000	-£214	(-£841 to £414)	0.25	-£220	(-£706 to £267)	0.19	-£77	(-£551 to £398)	0.38
£30,000	-£337	(-£1,135 to £461)	0.20	-£343	(-£1,067 to £381)	0.18	-£163	(-£817 to £492)	0.31
£50,000	-£583	(-£1799 to -£634)	0.17	-£589	(-£1,796 to £618)	0.17	-£334	(-£1,373 to £705)	0.26

Notes: *[a]Threshold value = cost per death avoided at 28 days; [b]NMB = net monetary benefit [mean (95% confidence interval)]; [c]PCE = probability of cost-effectiveness at given threshold.*

For peer review only

Table 7 Subgroup analysis: costs and outcome

		All (unapproached survivors excluded) N=4,468		Clinically significant episodes only N=3,010 (67%)		Clinically non-significant episodes only N=1,458 (33%)	
28-day NHS costs mean (95% CI)	Control	£9,604	(£9,243 to £9,967)	£9,456	(£8,991 to £9,922)	£9,451	(£8,786 to £10,116)
	RAPIDO	£9,572	(£9,204 to £9,441)	£9,618	(£9,134 to £10,102)	£9,047	(£8,399 to £9,696)
	Difference	-£33	(-£549 to £484)	£161	(-£469 to £791)	-£404	(-£1,308 to £501)
28-day survival mean (95% CI)	Control	0.78	(0.76 to 0.80)	0.78	(0.76 to 0.79)	0.80	(0.77 to 0.83)
	RAPIDO	0.77	(0.75 to 0.79)	0.77	(0.75 to 0.79)	0.78	(0.75 to 0.81)
	Difference	-0.01	(-0.04 to 0.01)	-0.01	(-0.04 to 0.02)	-0.02	(-0.06 to 0.02)

Table 8 Subgroup analysis: cost effectiveness at 28 days

Threshold value[a]	All (unapproached survivors excluded) N=4,468			Clinically significant episodes only N=3,010 (67%)			Clinically non-significant episodes only N=1,458 (33%)		
	NMB[b]	(95% CI)	PCE[c]	NMB	(95% CI)	PCE	NMB	(95% CI)	PCE
£5,000	-£29	(-£533 to £475)	0.46	-£206	(-£853 to £441)	0.27	£301	(-£629 to £1,231)	0.74
£10,000	-£91	(-£611 to £430)	0.37	-£250	(-£947 to £446)	0.24	£198	(-£803 to £1,199)	0.65
£20,000	-£214	(-£841 to £414)	0.25	-£339	(-£1,206 to £527)	0.22	-£9	(-£1,254 to £1,237)	0.49
£30,000	-£337	(-£1,135 to £461)	0.20	-£428	(-£1,521 to £664)	0.22	-£215	(-£1,785 to £1,355)	0.39
£50,000	-£583	(-£1799 to -£634)	0.17	-£606	(-£2,222 to £1,010)	0.23	-£627	(-£2,949 to £1,694)	0.30

[a]Threshold value = cost per death avoided at 28 days; [b]NMB = net monetary benefit [mean (95% confidence interval)]; [c]PCE = probability of cost-effectiveness at given threshold.

For peer review only - http://bmjopen.bmj.com/site/about/guidelines.xhtml

Discussion

Bloodstream infections are significant, prevalent clinical events associated with substantial morbidity, [1] mortality [17] and medical cost.[18] There are an estimated 1.2 million episodes of bloodstream infection and 157,000 associated deaths per year in Europe.[1] Identification of the aetiological agent is a critical step in the treatment of bloodstream infection.

We performed a within-trial economic evaluation of MALDI-TOF diagnostic technology for the rapid identification of the causative microbial agent in hospitalised patients with bloodstream infection, excluding patients with cultures not positive for growth. The trial's primary outcome of 28-survival was consistent with no difference between conventional and adjunctive MALDI-TOF identification. The economic analysis showed that the intervention was not likely to be cost-effective, measured using incremental cost and incremental 28-day survival. The subgroup analysis suggested that there were no differences in the cost-effectiveness of MALDI-TOF when accounting for the clinical significance of the infection.

However, the differences between arms were modest and associated with considerable uncertainty. It is important therefore to reflect on whether the use of this technology outside trial conditions might alter the conclusions of the within-trial evaluation. One consideration is that a higher MALDI-TOF throughput of machine-positive blood samples would reduce the overall cost per sample in the intervention arm. A value for the number of samples likely to be encountered in routine use was not included in the unit cost calculation in this study because of the exclusion criteria used in the trial: for example, it considered only samples from patients aged 18 and over. A reduction in the direct cost of MALDI-TOF would lower the intervention cost towards that of conventional diagnosis, but would not change patient outcomes.

Our economic evaluation did not calculate the cost of per-sample of conventional identification separately, since such costs are bundled into the ward stay costs and their inclusion would have amounted to double counting. By contrast, the intervention arm involved the use of MALDI-TOF in addition to conventional diagnosis, and hence the per-sample costs of MALDI-TOF are incremental to costs in the control arm. However, as MALDI-TOF has been increasingly adopted

For peer review only - http://bmjopen.bmj.com/site/about/guidelines.xhtml

in routine practice, experience shows that it is not, in fact, used as an adjunct to conventional approaches, but largely displaces them. In addition, its widespread adoption for use with samples from much more common infections (e.g. urinary tract infections) reduces its per-sample costs overall.

As a rough indication of the possible magnitudes of displacement that could be involved specifically for blood cultures, MALDI-TOF offered a usable identification of some 83% of first-bottle samples in the trial, and few of these samples would merit conventional identification in addition. Of the remaining 17% of samples, it is likely that, after further culture to produce colonial isolates, most would be successfully tested by MALDI-TOF without recourse to conventional biochemical methods.

Other considerations suggest that the incremental cost of MALDI-TOF compared to conventional identification could be smaller in "real-world" contexts than that identified in the RAPIDO trial. Longer term reductions in capital, labour and consumable inputs could not be measured within the period of trial-follow-up and are not reflected in the economic evaluation. For example, the MALDI-TOF process requires less physical space in the laboratory compared to conventional approaches, and a substantial long-term switch to the new technology would reduce the capital costs of microbiology laboratories, as would reductions in the cost of MALDI-TOF machines that may come from wider use and greater market competition. Changes in workflow using MALDI-TOF reduce the time required from laboratory staff to complete an identification, meaning that results can be supplied significantly faster to clinical staff on wards (a median of 35.6 hours after taking the blood sample using MALDI-TOF compared to 54.5 hours using conventional methods in the RAPIDO trial, $p<0.0001$ from a Wilcoxon test given violation of the proportional hazards assumption for this outcome).

Against this, it is important to note that ward costs accounted for 97% of all secondary care costs, and the absence of a significant effect of MALDI-TOF diagnosis in reducing the length of stay and improving survival is a central conclusion of the trial – and one that merits analysis in future research (discussed below). Without evidence for improvements in these outcomes,

For peer review only

For peer review only - http://bmjopen.bmj.com/site/about/guidelines.xhtml

reductions in the costs of MALDI-TOF diagnosis are plausible but may not materially alter the conclusions of our analysis.

Strengths and limitations

Strengths

To our knowledge, this is the first economic evaluation conducted alongside a randomised clinical trial evaluating MALDI-TOF for rapid identification as an addition to conventional microbiological and biochemical methods in bloodstream infection. There has been much observational evidence on the effects of MALDI-TOF technology on non-health outcomes such as time to identification, but there is a lack of trial-based evidence on the costs and cost-effectiveness of using MALDI-TOF in clinical contexts.[19]

The cost-effectiveness analysis was conducted on a large dataset offering individual-level cost data on thousands of patients. The costing of the intervention itself was supported by a time and motion observational study conducted at one of the trial sites, while ward and antimicrobial data were valued using nationally representative data sources.

Limitations

Mortality data was censored at 28 days, and information on ward type (by far the biggest cost driver) at seven days. We lacked the information necessary to examine longer-term costs and outcomes, albeit there is little reason to suspect that survival curves would diverge after 28 days to alter the primary survival outcome of the trial.

The cost analysis depended on a matching of the ward descriptions reported in the trial dataset to 'Service Descriptions' in NHS Reference Costs. This matching process was performed 'blind' to allocation, but the analysis of 28-day costs required an extrapolation from seven days to the earliest of death, discharge or the 28-day end-point. Unobserved changes in ward type after day 7 could change the estimated costs used in the base case, although no substantial difference was observed when comparing 28-day and 7-day analyses.

We did not have access to primary care records so our analysis was limited to a secondary care NHS perspective i.e. hospital resource use. In practice, because of the magnitude of per-day

For peer review only - http://bmjopen.bmj.com/site/about/guidelines.xhtml

ward costs, it is unlikely that accounting for other health system costs would have a material impact on our findings, given that the mortality outcome favoured conventional diagnosis.

Patients who were unable to consent for themselves and for whom no consultee was available comprised 19.5% (n=1,082) of the 5,550 patients in the analysis population, and no data beyond the mortality outcome, allocation, age and sex was available for them. However, apart from this group of patients, and the absence of ward-specific data between days 8 and 28, the amount of missing data in key cost drivers was limited.

The multiple imputation model estimated that these 1,082 patients had lower mean costs than the other 4,468 participants. Is this plausible? On the one hand, costs should be expected to diverge between the two groups given that the 1,082 patients who did not provide consent are all known to have survived for at least 28 days. Thus, the lower costs generated by the imputation model may reflect a population less acutely ill than the other participants. On the other hand, prolonged survival without discharge would give rise to higher costs than early death during the trial period.

These considerations complicate assessments of direction of the biases in the available case data. However, the conclusions that emerge from the multiple imputation results, the available case results, and the various sensitivity analyses are similar in identifying considerable uncertainty around the cost-effectiveness of MALDI-TOF in this patient group.

Finally, the economic evaluation was limited to identifying the cost-effectiveness of the intervention and did not identify the mechanisms that gave rise to the survival outcomes in the trial. Evidence from the analysis of secondary clinical outcomes in the RAPIDO trial indicates that time to provision of microbiological identification to the ward was significantly shorter in the RAPIDO arm and there was weak evidence of longer time to initiation of appropriate antimicrobial therapy in the RAPIDO arm (median 24.0 versus 13.0 hours, p=0.056 based on a Cox proportional hazards model). However, there was no significant difference between arms in other secondary outcomes: time to providing Gram stain and antimicrobial susceptibility results to the ward; time to resolution of fever (up to seven days) or discharge (up to 28 days); *C.*

For peer review only - http://bmjopen.bmj.com/site/about/guidelines.xhtml

difficile incidence (to 28 days); in-hospital antimicrobial consumption (to seven days) or the proportion of patients remaining on broad-spectrum therapy at seven days [2].

Future research

Future research could examine the mechanisms by which mortality outcomes may differ between MALDI-TOF and conventional diagnosis. Analysis of length of stay and survival in observational study designs in US contexts by Huang et al[20] and Perez et al [21] found beneficial effects of MALDI-TOF when used in conjunction with antimicrobial stewardship programmes, hinting at organisational changes that may be needed to exploit the faster identification offered by MALDI-TOF [22] . However, this is context-specific, as in other health systems, such as the NHS in which the present trial was conducted, bacteraemia consultation teams are routine and involved in care at an early stage.

Conclusion

Overall, the evidence from the RAPIDO trial suggests that the use of MALDI-TOF as an adjunct to conventional microbial identification is unlikely to offer value when its incremental costs are compared to 28-day incremental survival. It is plausible that the costs of MALDI-TOF in "real-world" routine use may well be lower than those measured during the RAPIDO trial, and savings can also be expected as it would displace much conventional testing.

For peer review only

For peer review only - http://bmjopen.bmj.com/site/about/guidelines.xhtml

Contributorship statement: PD and WH: conducted the economic analysis. PD wrote the first draft of this manuscript. PD, WH, KP, RR, MS, AMG: Reviewed, commented and edited the manuscript. AMG: Chief investigator for the RAPIDO trial.

Data statement: Permission to share individual-level data was not obtained from trial participants. Requests for summary level data should be directed to AMG.

Funding statement: This report summarises independent research funded by the National Institute for Health Research (NIHR) under its Programme Grant for Applied Research (PGfAR RP-PG-0707-10043). The views and opinions expressed in this report are those of the authors and do not necessarily reflect those of the National Institute for Health Research (NIHR), the NHS or the Department of Health. The funder had no role in the conduct of the study, the writing of the manuscript or the decision to submit it for publication.

Acknowledgements: We are very grateful to all patients, health care professionals, and NHS staff who contributed time and effort to make the RAPIDO trial possible. We are grateful to administrative staff at trial sites for support with participant recruitment, data entry and trial administration. The RAPIDO trial was designed and delivered in collaboration with Clinical Trials and Evaluation Unit Bristol (CTEU), a UKCRC Registered Clinical Trials Unit in receipt of National Institute for Health Research CTU support funding. RR acknowledges support from the NIHR Health Protection Research Unit in Behavioural Science and Evaluation at University of Bristol.

Competing interests statement: The authors declare no conflicts of interest.

Ethics: The RAPIDO trial was approved by the NRES Committee South West - Frenchay on 20 March 2012, reference 12/SW/003.

Informed consent: In order to initiate rapid diagnosis quickly, the study design required prompt randomisation when blood cultures flagged positive, so it was necessary to seek consent

For peer review only

For peer review only - http://bmjopen.bmj.com/site/about/guidelines.xhtml

retrospectively. Further details are provided in the main text and in references to the main trial publication.

For peer review only

References

1. Goto M, Al-Hasan MN. Overall burden of bloodstream infection and nosocomial bloodstream infection in North America and Europe. *Clin Microbiol Infect* 2013;19(6):501-09. doi: 10.1111/1469-0691.12195
2. MacGowan A, Grier S, Stoddart M, et al. Impact of rapid microbial identification on clinical outcomes in bloodstream infection: the RAPIDO randomised trial. *Clin Microbiol Infect* 2020 doi: https://doi.org/10.1016/j.cmi.2020.01.030
3. Timbrook TT, Morton JB, McConeghy KW, et al. The Effect of Molecular Rapid Diagnostic Testing on Clinical Outcomes in Bloodstream Infections: A Systematic Review and Meta-analysis. *Clinical infectious diseases : an official publication of the Infectious Diseases Society of America* 2017;64(1):15-23. doi: 10.1093/cid/ciw649 [published Online First: 2016/11/03]
4. Albur MS, Bowker K, Weir I, et al. Factors influencing the clinical outcome of methicillin-resistant Staphylococcus aureus bacteraemia. *European journal of clinical microbiology & infectious diseases : official publication of the European Society of Clinical Microbiology* 2012;31(3):295-301. doi: 10.1007/s10096-011-1310-2 [published Online First: 2011/06/15]
5. Laupland KB, Gregson DB, Church DL, et al. Incidence, risk factors and outcomes of Escherichia coli bloodstream infections in a large Canadian region. *Clinical microbiology and infection : the official publication of the European Society of Clinical Microbiology and Infectious Diseases* 2008;14(11):1041-7. doi: 10.1111/j.1469-0691.2008.02089.x [published Online First: 2008/12/02]
6. van Hal SJ, Jensen SO, Vaska VL, et al. Predictors of mortality in Staphylococcus aureus Bacteremia. *Clinical microbiology reviews* 2012;25(2):362-86. doi: 10.1128/cmr.05022-11 [published Online First: 2012/04/12]
7. Seifert H. The Clinical Importance of Microbiological Findings in the Diagnosis and Management of Bloodstream Infections. *Clin Infect Dis* 2009;48(Supplement 4):S238-S45. doi: http://dx.doi.org/10.1086/598188
8. Evans RN, Pike K, Rogers CA, et al. Modifiable healthcare factors affecting 28-day survival in bloodstream infection: a prospective cohort study. *BMC Infect Dis* 2020;20(1):545. doi: 10.1186/s12879-020-05262-6
9. Fournier P-E, Drancourt M, Colson P, et al. Modern clinical microbiology: new challenges and solutions. *Nature Reviews Microbiology* 2013;11(8):574–85. doi: http://dx.doi.org/10.1038/nrmicro3068
10. Curtis L, Burns A. Unit Costs of Health and Social Care 2019. Personal Social Services Research Unit. University of Kent, Canterbury, 2018.
11. Department of Health. Reference Costs 2012/13. London, 2013.
12. Joint Formulary Committee. British National Formulary (BNF): Pharmaceutical Press, 2012.
13. White IR, Royston P, Wood AM. Multiple imputation using chained equations: Issues and guidance for practice. *Stat Med* 2011;30(4):377-99. doi: 10.1002/sim.4067
14. Royston P, White IR. Multiple Imputation by Chained Equations (MICE): Implementation in Stata. *Journal of Statistical Software* 2011;45:1-20.
15. Faria R, Gomes M, Epstein D, et al. A Guide to Handling Missing Data in Cost-Effectiveness Analysis Conducted Within Randomised Controlled Trials. *Pharmacoeconomics* 2014;32(12):1157-70. doi: 10.1007/s40273-014-0193-3
16. Willan AR, Briggs AH, Hoch JS. Regression methods for covariate adjustment and subgroup analysis for non-censored cost-effectiveness data. *Health Econ* 2004;13(5):461-75. doi: 10.1002/hec.843

For peer review only - http://bmjopen.bmj.com/site/about/guidelines.xhtml

17. Bearman GML, Wenzel RP. Bacteremias: A Leading Cause of Death. *Arch Med Res* 2005;36(6):646-59. doi: http://dx.doi.org/10.1016/j.arcmed.2005.02.005
18. Kilgore M, Brossette S. Cost of bloodstream infections. *Am J Infect Control* 2008;36(10):S172.e1-S72.e3.
19. Dixon P, Davies P, Hollingworth W, et al. A systematic review of matrix-assisted laser desorption/ionisation time-of-flight mass spectrometry compared to routine microbiological methods for the time taken to identify microbial organisms from positive blood cultures. *Eur J Clin Microbiol Infect Dis* 2015;34(5):863-76. doi: 10.1007/s10096-015-2322-0
20. Huang AM, Newton D, Kunapuli A, et al. Impact of Rapid Organism Identification via Matrix-Assisted Laser Desorption/Ionization Time-of-Flight Combined With Antimicrobial Stewardship Team Intervention in Adult Patients With Bacteremia and Candidemia. *Clin Infect Dis* 2013;57(9):1237-45. doi: http://dx.doi.org/10.1093/cid/cit498
21. Perez KK, Olsen RJ, Musick WL, et al. Integrating Rapid Pathogen Identification and Antimicrobial Stewardship Significantly Decreases Hospital Costs. *Arch Pathol Lab Med* 2012;137(9):1247-54. doi: http://dx.doi.org/10.5858/arpa.2012-0651-OA
22. Kothari A, Morgan M, Haake D. Emerging Technologies for Rapid Identification of Bloodstream Pathogens. *Clin Infect Dis* 2014

For peer review only

For peer review only - http://bmjopen.bmj.com/site/about/guidelines.xhtml

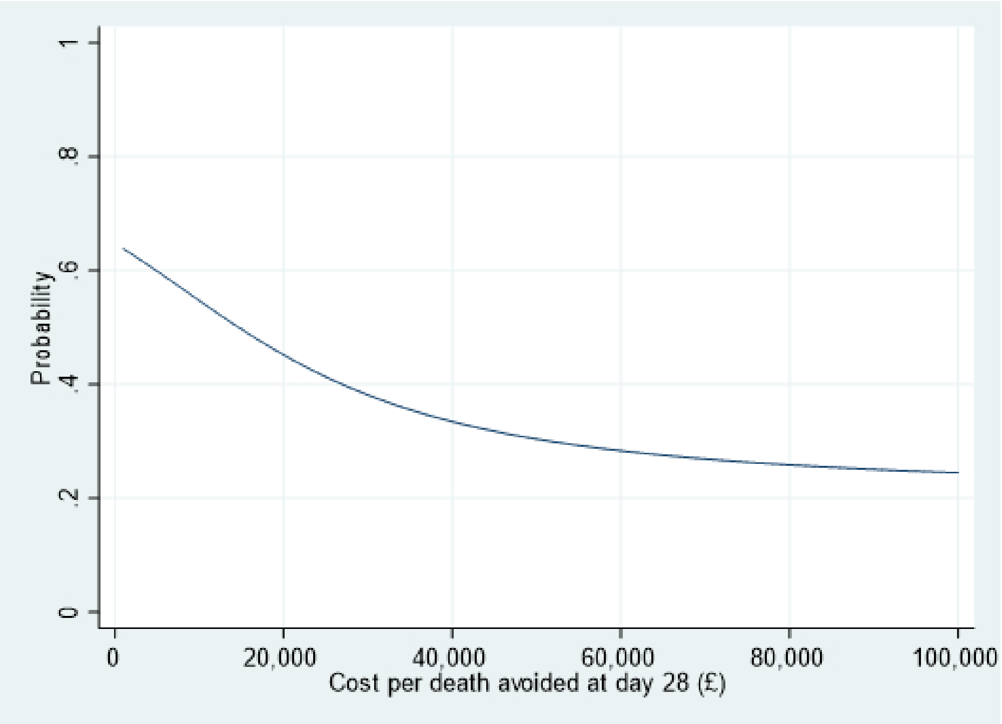

Cost-effectiveness acceptability curve for base case

SUPPLEMENTARY MATERIAL - Cost-effectiveness of rapid laboratory-based mass-spectrometry diagnosis of bloodstream infection: Evidence from the RAPIDO randomized controlled trial

Costing of hospital stays

The cost of ward stays was calculated from the 2012/13 NHS Reference Costs (1) and then inflated using Curtis and Burns (2) as described in main text.

Costing by ward speciality

Hospital admission was an inclusion criterion for the RAPIDO trial, but a small number of "non-admitted" ward-days could occur in cases where the diagnostic blood sample was taken in an outpatient or day-case setting shortly before admission to a more appropriate speciality. Therefore, all ward stays were costed as inpatients unless the ward speciality recorded in the dataset explicitly specified otherwise e.g. day-case surgery.

The ward specialities described in the dataset were mapped to their nearest equivalent Service Descriptions and currency codes in NHS Reference Costs as shown in Table A1. Where more than one Service Description or currency code applied to a ward speciality, the ward cost was calculated as an average of the unit costs of each Service Description or currency code, weighted by the number of bed-days reported for them in NHS Reference Costs. An example calculation is shown in Table A2.

The RAPIDO dataset did not include the number of organs supported in critical care so the cost of stays in high-dependency units (HDUs) and intensive therapy units (ITUs) was also calculated as a weighted average. The costs of a bed-day on an adult critical care unit for the different levels of organ support are listed in NHS Reference Costs; these costs were weighted by the number of bed-days reported for patients at each level of support to give an average cost for use in analysis.

For peer review only

Table A1 Matching of RAPIDO ward specialities to NHS Reference Cost categories

**RAPIDO ward speciality description**	**NHS Reference Cost 'Service Description'**	**NHS Reference Cost 'service code' or 'currency description'[a,b]**
Medical: Acute medical admissions and pre-admissions	Accident and Emergency	Admitted codes only[c]
Medical: Cardiology / cardiovascular / coronary	Cardiology	320
Medical: Care of the Elderly	Geriatric Medicine	430
Medical: Dermatology / rheumatology	Dermatology AND Rheumatology	410[R] AND 330[D]
Medical: Diabetes / endocrinology	Diabetic Medicine AND Endocrinology	307[D] AND 302[E]
Medical: Gastroenterology / gastrology / liver	Gastroenterology AND Hepatology	301 [G] and 306[H]
Medical: General medical (no declared speciality)	General Medicine	300
Medical: Haematology / oncology	Clinical haematology AND Clinical oncology	303[H] and 800[O]
Medical: Infectious disease / travel medicine	Infectious Diseases	350
Medical: Nephrology / renal / dialysis	Nephrology	361
Medical: Neurology / neurosciences / neuromedical	Neurology	400
Medical: Palliative	Palliative Medicine	315
Medical: Respiratory	Respiratory Medicine	328
Medical: Stroke	Stroke Medicine	340
ITU/HDU[d]: General (not specified as surgical, medical or specialist)	Adult Critical Care Unit	Critical care currencies
ITU/HDU: General medical	N/A[e]	N/A
ITU/HDU: General surgical	Adult Critical Care Unit	Critical care currencies
ITU/HDU: Cardiac	Adult Critical Care Unit	Critical care currencies
ITU/HDU: Neurology/neurosurgery	Adult Critical Care Unit	Critical care currencies
ITU/HDU: Renal	N/A	N/A
ITU/HDU: Theatre recovery areas	N/A	N/A
Surgery: Admissions / pre-admissions units	General surgery	100
Surgery: Cardiothoracic / thoracic	Cardiothoracic surgery AND Thoracic surgery	170[C] and 173[T]
Surgery: Ear, nose, throat, oral & maxillo-facial, and opthalmic units	All surgical codes[f]	Inpatient codes only[c]
Surgery: General including GI, breast, vascular	General surgery	100

For peer review only

Surgery: Neurosurgery	Neurosurgery	150
Surgery: Orthopaedic / trauma	Trauma & Orthopaedics	110
Surgery: Plastics / burns	Plastic surgery	160
Surgery: Short stay and daycase units	General surgery	100, day cases only
Surgery: Urology / renal	All surgical codes[f]	Inpatient codes only[c]
Other: A&E, emergency assessment, fracture clinics and related units	Accident and Emergency	Admitted codes only[c]
Other: Imaging, diagnostics and telemetry	N/A	N/A
Other: Obstetrics & gynaecology	Obstetrics	501
Other: Psychology / psychiatry / mental health	N/A	N/A
Other: Services - not medical, surgical or HDU/ITU, and not listed elsewhere	"Other Procedures or Health Care Problems"	Inpatient codes only[c]

Notes to Table A1:

[a] If there were separate codes for adults and children, only adult codes were used.

[b]If multiple NHS Service Descriptions or Currency Codes applied, costs were weighted by the number of bed-days occupancy for each Description or Code (or, in critical care, number of organs supported) reported in NHS Reference Costs.

[c] Only 'Inpatient' or 'Admitted' codes were used in these cases as all RAPIDO patients were admitted before or soon after taking of the diagnostic blood sample.

[d]HDU/ITU = critical care specialities (high dependency and intensive therapy units), costs weighted by number of bed-days occupancy for each number of organs supported in critical care.

[e] N/A = Not applicable – not costed as no observations in the trial.

[f]A weighted average across all admitted surgical codes was used for surgical specialities that were not listed specifically in NHS Reference Costs. Costing by length of stay

Hospitals in the NHS are paid according to patients' length of stay, with different levels of payment being made according to whether each bed-day of the stay is an 'inlier' bed-day or an excess (or 'outlier') bed-day. These payments constitute 'costs' from an NHS system perspective and we used them to cost hospital stays.

The total costs of the patients' stays in each arm of the trial depend on their whole ward history from the date of admission, not from the date of randomisation - although, clearly, differences due to the RAPIDO intervention could only accrue after randomisation. The total hospital cost could not be calculated because, although the date of admission was known, it was not known for how long patients had been on particular wards before the diagnostic blood samples were taken.

The distinction between an inlier bed-day and an excess bed-day is defined by reference to a trim point, a figure which is intended to capture the upper end of the range of lengths of stay expected for a given currency code. It is calculated as the (upper quartile of length of stay) + 1.5 × (interquartile range of length of stay). In a long stay, days after the trim point are counted as excess bed-days, while days up to the trim point are counted as inliers.

For inlier costs, national average unit costs and average length of stay (number of days) per Finished Consultant Episode are reported. A Finished Consultant Episode is a completed episode of treatment

For peer review only

received by a patient under the care of one consultant. Dividing the mean nationwide unit cost for a given currency code by the mean nationwide number of inpatient days for a patient admitted under that currency code gives its estimated per-day cost. To obtain the weighted cost for each currency code, we multiply the per-day cost by the share of that currency code in the total bed-days of all currency codes in each Service Description. The sum of these figures is the weighted average per-day cost for either elective inpatient or non-elective inpatients receiving treatment under that Service Description. Unit costs per excess bed day are calculated in a similar manner.

Elective and non-elective stays

The RAPIDO dataset did not include information to classify each day of each patient's stay in a ward as elective or non-elective. It is likely that a high proportion of ward admittances after day 0 of the trial were non-elective since they followed a presumed diagnosis (or, at least, clear suspicion) of bloodstream infection, as evidenced by the taking of a blood sample for culture. However, it is possible that the infection developed during an elective admission, so ward costs – particularly on day 0 – might in fact be charged as elective. We therefore included both elective and non-elective costs in our calculations, by weighting the average cost of all currency codes in each category (elective and non-elective) by their respective shares in bed-days.

A post hoc analysis examined the sensitivity of unit costs to the exclusion of elective care. The effect on unit costs of this exclusion was modest. In some cases, unit costs do not distinguish between elective and non-elective (e.g. for critical care) and the exclusion had no effect, and in other cases the effect of excluding elective admissions was to reduce unit costs by approximately 0.1% to 1%. We consider that including elective care in the calculation of unit costs in general is a conservative approach, and one appropriate to our base-case analysis. We therefore did not re-run this analysis using the very slightly lower unit costs that would have arisen under the exclusion of elective care.

Example: calculation of per-day cost for a 'General Medical' ward stay

The steps involved in the calculation of unit costs for the 'General Medicine' Service Description are set out in Table A2.

Table A2 Example calculation of weighted per-day cost associated with the 'General Medicine' Service Description in 2012/13

Item	Quantity or share of total	Calculation
Number of bed-days[a]		
National total bed-days per year: Elective Inpatient excluding short-stay (EI)	96,339	A
National total bed-days per year: Non-Elective Inpatient (NEI)	6,398,877	B
National total bed-days per year: Non-Elective Inpatient Short-stay (NEI-S)	1,545,596	C
National total bed-days per year: Inpatients (EI + NEI + NEI-S)	8,040,812	D=(A+B+C)
Share of bed-days		

For peer review only

% of days that relate to EI	1.20%	E=A/D
% of days that relate to NEI	79.58%	F=B/D
% of days that relate to NEI-S	19.22%	G=C/D
Averaging over inlier/outlier bed-days and currency codes		
Weighted[b] average per-day cost: EI	£456.18	H[b]
Weighted[b] average per-day cost: NEI	£327.48	I[b]
Weighted[b] average per-day cost: NEI-S	£434.19	J[b]
Contributions of elective, non-elective and short stays		
Weighted[c] EI cost per day	£5.47	K=H × E
Weighted[c] NEI cost per day	£260.61	L=F × I
Weighted[c] NEI-S cost per	£83.46	M=G × J
Estimated mean cost per day for RAPIDO analysis		
Cost of a General Medical ward-day for RAPIDO analysis i.e. weighted average of EI, NEI and NEI-S per day costs	£349.53	K+L+M

[a]'Bed-days' here captures both bed days and excess bed days. [b]These figures are calculated as the average of bed-day-weighted sum of costs for inlier bed-days and excess bed-days across all currency codes within the 'General Medicine' service description, weighted by proportion of bed-days from each currency code, as described in the text. [c]These figures are weighted by the proportion of bed-days from each type of inpatient stay (elective, non-elective, and non-elective short-stay).

Costing of MALDI-TOF spectrometry

Data from published literature and confidential information provided by one study centre was used to estimate a mean per blood-sample cost of diagnosis using MALDI-TOF technology at 2012/13 prices and then subsequently inflated.

At this centre, 4,303 machine-positive adult blood samples were recorded during the whole study period, equating to 2,061 per year. In routine use of the technology, all of these would have been tested by MALDI-TOF but fewer were actually tested during the RAPIDO trial. Only 3,153 flagged positive during study hours (=1510/year) and, with 1:1 randomisation, only approximately half of those (755) would have been allocated to MALDI-TOF diagnosis.

We estimated the proportion of total MALDI-TOF cost attributable to use for positive blood cultures as 12% based on two considerations. First, approximately 10-15% of all microbiology laboratory requests for 'culture and sensitivity' related to blood samples. Second, at this centre, organisms from blood accounted for approximately 12% of all organisms identified.

We used these figures to calculate the capital, operating, labour and consumable costs used per positive sample as set out in Table A3. Salary costs and information concerning on costs and overheads were taken from Curtis (2013).(3)

For peer review only

Table A3 Calculation of unit cost of MALDI-TOF identification in 2012/13 prices

Item	Mean value	Comment / data source
Capital-related costs		
Capital cost for new MALDI-TOF machine	£130,000	Published literature, catalogue prices, confidential information from one study centre
Economic life used for depreciation calculations	10 years	Published literature, catalogue prices, confidential information from one study centre
Annual capital charge per blood sample	£0.76	
Maintenance costs		
Annual non-reagent maintenance contract (13% of capital cost)	£16,900	Published literature, confidential information from one study centre
Maintenance cost per blood sample	£0.98	
Consumable costs		
Consumables cost per blood sample, as used in RAPIDO protocol	£0.17	Trial protocol, catalogue data, information from one study centre
Labour costs, including on-costs and overheads		
Band 5 salary	£62,927	Staff grades at one study centre, published NHS payscales, Curtis (2013) for information on oncosts and overhead
Band 6 salary	£76,569	Staff grades at one study centre; published NHS payscales; information on on-costs and overheads from Curtis (2013).(3)
Labour cost per sample	£4.17	...assuming that each grade contributes equally to processing of all blood samples
Total cost		
Total cost per machine-positive blood sample analysed with MALDI-TOF	£6.08	

For peer review only

CONSORT diagram of patient numbers

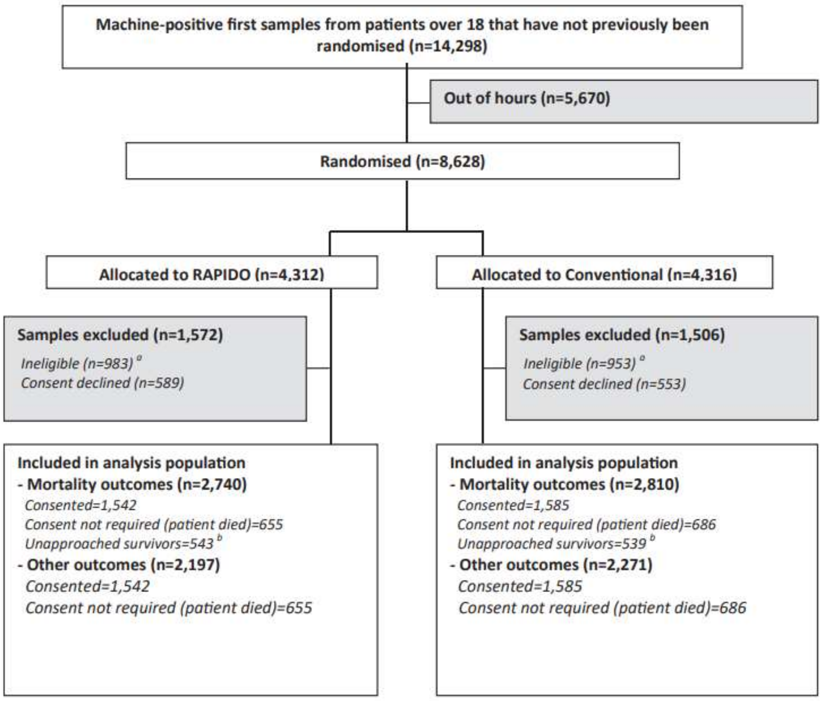

Notes to figure: Flow of patients. a Ineligible samples include 111 rapid diagnosis and 125 conventional samples that were randomized in error, and 872 rapid diagnosis and 828 conventional that were randomized correctly but met postrandomization exclusion criteria. b Unapproached survivors are eligible patients who could not be approached for consent, usually because of lack of capacity and inability to identify a consultee. They are included in mortality analysis (only) as 28-day survivors.

Costs and results in 2012/13 price levels

Table A4 Costs in available cases

Mean cost	Control (N=2,271)	RAPIDO (N=2,197)	Difference (95% CI) [a]
Intervention cost	-	£6	+£6
Antimicrobial cost	£247	£265	+£18 (-£6 to £41)
7-day ward costs	£3,448	£3,404	-£44 (-£165 to £77)
Total 7-day costs	£3,695	£3,675	-£20 (-£148 to £108)
28-day ward costs	£8,451	£8,412	-£39 (-£505 to £427)
Total 28-days costs	£8,698	£8,682	-£15 (-£487 to £456)

Note: *[a] Confidence intervals around mean differences calculated from unadjusted linear regression*

Cost-effectiveness and sensitivity analysis

In the base-case imputed analysis, estimated mean costs per patient were lower in the RAPIDO arm (mean difference -£114; 95% CI:-£710 to £482), and the proportion of patients alive at day 28 was also lower (81.4% vs 82.3%, see Table A5). Ward costs, including the costs of conventional microbiological testing, constituted 97% of total costs in each arm. Most of the remaining 3% of total cost was attributable to antimicrobial costs. The estimated per-patient cost of diagnosis using MALDI-TOF (£6.08) constituted a negligible proportion of overall per-patient mean costs in the intervention arm.

Table A5 Costs and outcome: base-case analysis with imputation (N=5,550)

	Control	RAPIDO	Difference (95% CI)
Mean 28-day NHS costs	£ 7,485	£7,371	-£114 (-£710 to £482)
28-day survival[a]	0.823	0.814	-0.009 (-0.029 to 0.011)

Note: *[a]Survival measured as the proportion of patients alive at day 28.*

Table A6 Cost-effectiveness: base-case analysis with imputation (N=5,550)

Threshold[a]	Net monetary benefit (95% CI)	Probability of cost-effectiveness
£5,000	£71 (-£519 to £661)	0.59
£10,000	£28 (-£579 to £636)	0.54
£20,000	-£57 (-£745 to £630)	0.44
£30,000	-£143 (-£954 to £668)	0.36
£50,000	-£315 (-£1,443 to £814)	0.29

Note: *[a]Threshold value = 28-day cost per death avoided at 28 days.*

The probability of the RAPIDO intervention being cost-effective declines with increasing threshold values of cost per death avoided at 28 days, as shown in Figure A1.

For peer review only

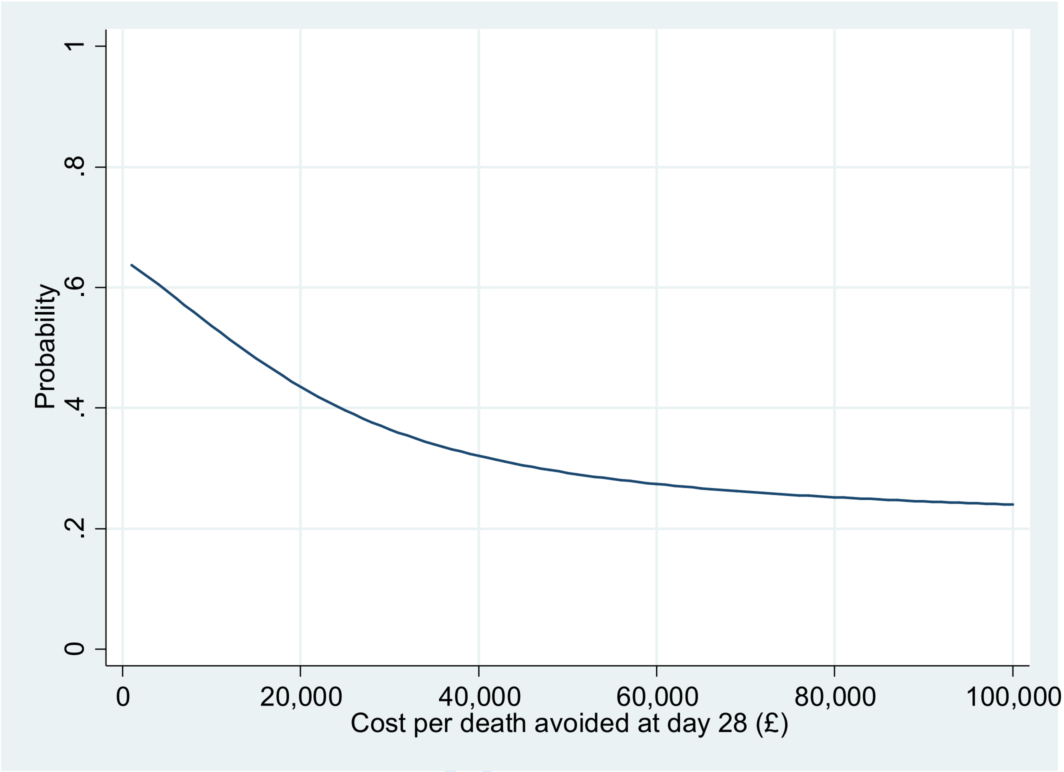

Figure A1 Cost-effectiveness acceptability curve for base case

Table A7 and A8 report the results of the various sensitivity analyses. These analyses, expressed as net monetary benefit (with associated 95% confidence intervals), do not differ substantially from the base-case results. Estimating costs at seven rather than 28 days did not alter the overall cost-effectiveness conclusions.

Subgroup analysis

Table A9 and Table A10 present the results of the subgroup analysis comparing clinically significant and clinically non-significant episodes of bloodstream infection. Statistical tests for interaction showed no evidence of a subgroup effect (p-value for interaction in the cost seemingly unrelated regression equation=0.32, p-value in the outcome seemingly unrelated regression equation =0.66), and estimates of difference between Conventional and RAPIDO diagnosis in both outcome and costs were broadly similar for the clinically significant and clinically non-significant subgroups.

Table A7 Sensitivity analysis: costs and outcome

		Excluding unapproached survivors, N=4,468, cost at 28 days		Excluding unapproached survivors, N=4,468, cost at 7 days		Including unapproached survivors, N=5,550, cost at 7 days	
NHS costs mean (95% CI)	Control	£8,705	(£8,376 to £9,033)	£3,697	(£3,608 to £3,786)	£3,324	(£3,098 to £3,598)
	RAPIDO	£8,675	(£8,341 to £9,009)	£3,673	(£3,583 to £3,763)	£3,238	(£3,094 to £3,619)
	Difference	-£30	(-£498 to £439)	-£24	(-£151 to £103)	-£86	(-£324 to £153)
28-day survival mean (95% CI)	Control	0.78	(0.76 to 0.80)	0.78	(0.76 to 0.80)	0.82	(0.81 to 0.84)
	RAPIDO	0.77	(0.75 to 0.79)	0.77	(0.75 to 0.79)	0.81	(.80 to 0.83)
	Difference	-0.01	(-0.04 to 0.01)	-0.01	(-0.04 to -0.01)	-0.01	(-0.03 to 0.01)

Table A8 Sensitivity analysis: cost effectiveness

	Excluding unapproached survivors, N=4,468, cost at 28 days			Excluding unapproached survivors, N=4,468, cost at 7 days			Including unapproached survivors, N=5,550, cost at 7 days		
Threshold[a]	NMB	(95% CI)	PCE[b]	NMB	(95% CI)	PCE	NMB	(95% CI)	PCE
£50,000	-£32	(-£489 to £425)	0.45	-£38	(-£199 to £124)	0.32	£43	(-£209 to £295)	0.63
£10,000	-£94	(-£572 to £385)	0.35	-£99	(-£356 to £158)	0.23	£0	(-£305 to -£305)	0.50
£20,000	-£217	(-£814 to £381)	0.24	-£222	(-£707 to £263)	0.18	-£86	(-£548 to £377)	0.36
£30,000	-£340	(-£1,117 to £438)	0.20	-£345	(-£1,069 to £379)	0.17	-£171	(-£817 to £474)	0.30
£50,000	-£586	(-£1,793 to £622)	0.17	-£591	(-£1,799 to £616)	0.17	-£343	(-£1,376 to £691)	0.26

Notes: *[a]Threshold value = cost per death avoided at 28 days; [b]NMB = net monetary benefit [mean (95% confidence interval)]; [c]PCE = probability of cost-effectiveness at given threshold.*

For peer review only

Table A9 Subgroup analysis: costs and outcome

		All (unapproached survivors excluded) N=4,468		Clinically significant episodes only N=3,010 (67%)		Clinically non-significant episodes only N=1,458 (33%)	
28-day NHS costs mean (95% CI)	Control	£8,705	(£8,376 to £9,033)	£8,570	(£8,148 to £8,992)	£8,565	(£7,962 to £9,168)
	RAPIDO	£8,675	(£8,341 to £9,009)	£8,716	(£8,278 to £9,155)	£8,199	(£7,611 to £8,787)
	Difference	-£30	(-£498 to £439)	£146	(-£425 to £717)	-£365	(-£1,186 to £454)
28-day survival mean (95% CI)	Control	0.78	(0.76 to 0.80)	0.78	(0.76 to 0.79)	0.80	(0.77 to 0.83)
	RAPIDO	0.77	(0.75 to 0.79)	0.77	(0.75 to 0.79)	0.78	(0.75 to 0.81)
	Difference	-0.01	(-0.04 to 0.01)	-0.01	(-0.04 to 0.02)	-0.02	(-0.06 to 0.02)

Table A10 Subgroup analysis: cost effectiveness at 28 days

Threshold value[a]	All (unapproached survivors excluded) N=4,468			Clinically significant episodes only N=3,010 (67%)			Clinically non-significant episodes only N=1,458 (33%)		
	NMB[b]	(95% CI)	PCE[c]	NMB	(95% CI)	PCE	NMB	(95% CI)	PCE
£50,000	-£32	(-£489 to £425)	0.45	-£191	(-£780 to £390)	0.26	£263	(-£585 to £1,110)	0.73
£10,000	-£94	(-£572 to £385)	0.35	-£235	(-£879 to £409)	0.24	£160	(£765 to £1,085)	0.63
£20,000	-£217	(-£814 to £381)	0.24	-£324	(-£1,149 to £501)	0.22	-£47	(-£1,231 to £1,138)	0.47
£30,000	-£340	(-£1,117 to £438)	0.20	-£413	(-£1,472 to £647)	0.22	-£253	(-£276 to -£230)	0.37
£50,000	-£586	(-£1,793 to £622)	0.17	-£591	(-£2,814 to £1,003)	0.23	-£665	(-£700 to -£631)	0.28

[a]Threshold value = cost per death avoided at 28 days; [b]NMB = net monetary benefit [mean (95% confidence interval)]; [c]PCE = probability of cost-effectiveness at given threshold.

References

For peer review only - http://bmjopen.bmj.com/site/about/guidelines.xhtml

1. **Department of Health** (2013) Reference Costs 2012/13. London.

2. **Curtis L & Burns A** (2018) Unit Costs of Health and Social Care 2019. Personal Social Services Research Unit. University of Kent, Canterbury.

3. **Curtis L** (2013) *Unit Costs of Health and Social Care 2013*. Canterbury: Personal Social Services Research Unit.

For peer review only

CHEERS checklist—Items to include when reporting economic evaluations of health interventions

Section/item	Item No	Recommendation	Reported on page No/ line No
**Title and abstract**			
Title	1	Identify the study as an economic evaluation or use more specific terms such as "cost-effectiveness analysis", and describe the interventions compared.	P1
Abstract	2	Provide a structured summary of objectives, perspective, setting, methods (including study design and inputs), results (including base case and uncertainty analyses), and conclusions.	P1
**Introduction**			
Background and objectives	3	Provide an explicit statement of the broader context for the study.	P5-6
		Present the study question and its relevance for health policy or practice decisions.	P5-6
**Methods**			
Target population and subgroups	4	Describe characteristics of the base case population and subgroups analysed, including why they were chosen.	P5-6
Setting and location	5	State relevant aspects of the system(s) in which the decision(s) need(s) to be made.	P5-6
Study perspective	6	Describe the perspective of the study and relate this to the costs being evaluated.	P5
Comparators	7	Describe the interventions or strategies being compared and state why they were chosen.	P5
Time horizon	8	State the time horizon(s) over which costs and consequences are being evaluated and say why appropriate.	P7
Discount rate	9	Report the choice of discount rate(s) used for costs and outcomes and say why appropriate.	N/A
Choice of health outcomes	10	Describe what outcomes were used as the measure(s) of benefit in the evaluation and their relevance for the type of analysis performed.	N/A

For peer review only

Section/item	Item No	Recommendation	Reported on page No/ line No
Measurement of effectiveness	11a	*Single study-based estimates:* Describe fully the design features of the single effectiveness study and why the single study was a sufficient source of clinical effectiveness data.	P25-7
	11b	*Synthesis-based estimates*: Describe fully the methods used for identification of included studies and synthesis of clinical effectiveness data.	N/A
Measurement and valuation of preference based outcomes	12	If applicable, describe the population and methods used to elicit preferences for outcomes.	N/A
Estimating resources and costs	13a	*Single study-based economic evaluation:* Describe approaches used to estimate resource use associated with the alternative interventions. Describe primary or secondary research methods for valuing each resource item in terms of its unit cost. Describe any adjustments made to approximate to opportunity costs.	P7-9
	13b	*Model-based economic evaluation:* Describe approaches and data sources used to estimate resource use associated with model health states. Describe primary or secondary research methods for valuing each resource item in terms of its unit cost. Describe any adjustments made to approximate to opportunity costs.	N/A
Currency, price date, and conversion	14	Report the dates of the estimated resource quantities and unit costs. Describe methods for adjusting estimated unit costs to the year of reported costs if necessary. Describe methods for converting costs into a common currency base and the exchange rate.	P7-9
Choice of model	15	Describe and give reasons for the specific type of decision-analytical model used. Providing a figure to show model structure is strongly recommended.	N/A

For peer review only

Section/item	Item No	Recommendation	Reported on page No/ line No
Assumptions	16	Describe all structural or other assumptions underpinning the decision-analytical model.	N/A
Analytical methods	17	Describe all analytical methods supporting the evaluation. This could include methods for dealing with skewed, missing, or censored data; extrapolation methods; methods for pooling data; approaches to validate or make adjustments (such as half cycle corrections) to a model; and methods for handling population heterogeneity and uncertainty.	P9-11
**Results**			
Study parameters	18	Report the values, ranges, references, and, if used, probability distributions for all parameters. Report reasons or sources for distributions used to represent uncertainty where appropriate. Providing a table to show the input values is strongly recommended.	P11-12
Incremental costs and outcomes	19	For each intervention, report mean values for the main categories of estimated costs and outcomes of interest, as well as mean differences between the comparator groups. If applicable, report incremental cost-effectiveness ratios.	P11-12
Characterising uncertainty	20a	*Single study-based economic evaluation:* :Describe the effects of sampling uncertainty for the estimated incremental cost and incremental effectiveness parameters, together with the impact of methodological assumptions (such as discount rate, study perspective).	P12-16
	20b	*Model-based economic evaluation:* Describe the effects on the results of uncertainty for all input parameters, and uncertainty related to the structure of the model and assumptions.	N/A
Characterising heterogeneity	21	If applicable, report differences in costs, outcomes, or cost-effectiveness that can be explained by variations between subgroups of patients with different baseline characteristics or	N/A

For peer review only

Section/item	Item No	Recommendation	Reported on page No/ line No
		other observed variability in effects that are not reducible by more information.	
**Discussion**			
Study findings, limitations, generalisability, and current knowledge	22	Summarise key study findings and describe how they support the conclusions reached. Discuss limitations and the generalisability of the findings and how the findings fit with current knowledge.	P17-21
**Other**			
Source of funding	23	Describe how the study was funded and the role of the funder in the identification, design, conduct, and reporting of the analysis. Describe other non-monetary sources of support.	P1
Conflicts of interest	24	Describe any potential for conflict of interest of study contributors in accordance with journal policy. In the absence of a journal policy, we recommend authors comply with International Committee of Medical Journal Editors recommendations.	P2

For consistency, the CHEERS statement checklist format is based on the format of the CONSORT statement checklist

For peer review only
